# Non-equilibrium critical dynamics of bursts in *θ* and *δ* rhythms as fundamental characteristic of sleep and wake micro-architecture

**DOI:** 10.1371/journal.pcbi.1007268

**Published:** 2019-11-14

**Authors:** Jilin W. J. L. Wang, Fabrizio Lombardi, Xiyun Zhang, Christelle Anaclet, Plamen Ch. Ivanov

**Affiliations:** 1 Keck Laboratory for Network Physiology, Department of Physics, Boston University, Boston, Massachusetts, United States of America; 2 Institute of Science and Technology Austria, A-3400 Klosterneuburg, Austria; 3 Department of Neurobiology, University of Massachusetts Medical School, Worcester, Massachusetts, United States of America; 4 Department of Neurology, Division of Sleep Medicine, Harvard Medical School and Beth Israel Deaconess Medical Center, Boston, Massachusetts, United States of America; 5 Harvard Medical School and Division of Sleep Medicine, Brigham and Women’s Hospital, Boston, Massachusetts, United States of America; University of Pittsburgh, UNITED STATES

## Abstract

Origin and functions of intermittent transitions among sleep stages, including short awakenings and arousals, constitute a challenge to the current homeostatic framework for sleep regulation, focusing on factors modulating sleep over large time scales. Here we propose that the complex micro-architecture characterizing the sleep-wake cycle results from an underlying non-equilibrium critical dynamics, bridging collective behaviors across spatio-temporal scales. We investigate *θ* and *δ* wave dynamics in control rats and in rats with lesions of sleep-promoting neurons in the parafacial zone. We demonstrate that intermittent bursts in *θ* and *δ* rhythms exhibit a complex temporal organization, with long-range power-law correlations and a robust duality of power law (*θ*-bursts, active phase) and exponential-like (*δ*-bursts, quiescent phase) duration distributions, typical features of non-equilibrium systems self-organizing at criticality. Crucially, such temporal organization relates to anti-correlated coupling between *θ*- and *δ*-bursts, and is independent of the dominant physiologic state and lesions, a solid indication of a basic principle in sleep dynamics.

## Author summary

Sleep exhibits intermittent transitions among sleep stages and short awakenings, with continuous fluctuations within stages that trigger micro-states and brief arousals. Despite the established association between dominant brain rhythms and physiologic states, the nature and dynamics of sleep-wake and sleep-stage transitions remain not understood. Homeostatic models of sleep regulation at ultradian and circadian scales do not address empirical observations of spontaneous transitions in sleep micro-architecture, and do not account for the emergent complex structure of sleep stages and arousals, and the related dynamics of bursts in cortical rhythms. Empirical observations of intrinsic bursts in cortical activity, and corresponding intermittent transitions in sleep micro-architecture, raise the hypothesis that non-equilibrium critical dynamics underlie sleep regulation at short time scales. We analyze *θ* and *δ* cortical rhythms in control rats and rats with lesions in the parafacial zone, which plays a significant role in the regulation of slow-wave sleep. The results demonstrate that critical dynamics underlie cortical activation during sleep and wake, and lay the foundation for a new paradigm, considering sleep micro-architecture as result of a non-equilibrium process and self-organization among neuronal assemblies to maintain a critical state, in contrast to the homeostasis paradigm of sleep regulation at large time scales.

## Introduction

The brain’s ability to adapt and perform complex functions crucially depends on the cooperation of assemblies of neurons across multiple spatial and temporal scales. Cortical rhythms represent one of the most fascinating collective phenomena emerging from the self-organized synchronous activity of large neuronal populations, and are consistently associated with complex brain functions and distinct physiologic states such as sleep and wake [1, 2]. Different brain rhythms characterize distinct phases of the sleep-wake cycle. During deep NREM sleep, brain dynamics are generally dominated by *δ* rhythm, low-frequency high-amplitude oscillations referred to as slow-wave activity [3]. Such slow-wave oscillations result from the synchronized activity of cortical neurons alternating between ‘up’ and ‘down’ states. In the up state, cortical neurons are depolarized, i.e. their membrane potential is closer to the firing threshold, and they fire in bursts of close-in-time action potentials. Conversely, in the down state, cortical neurons are hyperpolarized, i.e. their membrane potential is lower than the resting potential, and stay mostly silent [4]. Slow-wave activity can be modulated by the thalamus via thalamo-cortical neurons [5], and is enhanced by chemogenetic activation of GABAergic neurons in the parafacial zone (PZ) [6], while activation of cholinergic neurons in the basal forebrain results in significant decrease of slow-wave activity [7, 8]. In contrast to NREM sleep, REM sleep and arousals/wake state are characterized by desychronized and localized cortical rhythms of higher frequency and lower amplitude, such as *α* waves in resting humans and *θ* waves in rodents [9]. The *θ* rhythm can be generated both in the cortex and in the hippocampus. During wakefulness, cortical *θ* rhythm is driven by excitatory inputs from cholinergic, histaminergic, orexinergic, dopaminergic and noradrenergic sub-cortical neurons [10]. During REM sleep, hippocampal *θ* rhythm is driven by GABAergic inputs from the medial septum [11].

Despite the established association between dominant brain rhythms and emergent physiologic states, the nature and dynamics of sleep-wake and sleep-stage transitions remain not understood. Indeed, sleep periods exhibit numerous abrupt transitions among sleep stages and short awakenings, with continuous fluctuations within sleep stages triggering micro-states and brief arousals [12–14]. Such transient behavior is typically observed for a class of physical systems exhibiting self-organization and characterized by (i) multi-component nonlinear feedback interactions; (ii) high susceptibility and responsiveness to perturbations; (iii) non-equilibrium output dynamics with continuous fluctuations over a broad range of time scales; and (iv) maintaining a critical state where alternating active and quiescent phases co-exist [15–17]. This is a challenge to the current conceptual framework for sleep regulation, which is based on homeostasis, considers sleep as an equilibrium process, and focuses on factors modulating sleep over large time scales, such as homeostatic sleep drive, sleep propensity and inertia, ultradian and circadian rhythms [9, 18]. Models developed within the homeostasis paradigm have successfully accounted for consolidated sleep and wakefulness over time scales of hours and days, reproducing homeostatic, ultradian and circadian influences [9]. Further, a flip-flop switching mechanism involving mutually inhibitory interactions between sleep- and wake-promoting neuronal assemblies distributed across brain areas [19–21], and regulated by slow homeostatic factors such as adenosine and nitric oxide [9], has been proposed to explain the transition from stable sleep to stable wakefulness. However, the mechanism responsible for turning the switch on and off, the dynamic characteristics and temporal organization of these transitions remain unclear. Moreover, existing homeostatic models of sleep regulation (i) do not address empirical observations of transient behaviors at scales of seconds and minutes—an intrinsic sleep micro-architecture on time scales much shorter than consolidated sleep and wake states which last several hours, and sleep-stage episodes lasting from many minutes to an hour; and (ii) do not account for the emergent complex structure of sleep stage and arousal/brief wake transitions and the related micro-architecture of bursts in cortical brain waves activity. The intrinsic fluctuations in activity of brain rhythms in response to nonlinear feedback interactions among multi-component sleep- and wake-promoting neuronal pathways, the high susceptibility to abrupt transitions and the resulting complex temporal organization of sleep micro-architecture at scales of seconds and minutes rise the hypothesis that non-equilibrium critical dynamics may underlie sleep regulation at short time scales, in coexistence with the well-established homeostatic behavior at large time scales.

The traditional description of sleep stages in terms of dominant cortical rhythms indeed provides only an ‘average’, coarse-grained phenomenological picture that does not reflect the complex dynamics of sleep microstates and brief arousals. Current guidelines for sleep-stage classification do not consider the role of dynamical interactions among brain rhythms [3], although these interactions are potentially an integral feature of the collective neuronal activity driving physiologic states and related sleep-stage transitions [22–25]. Furthermore, the relation between emergent cortical brain-waves dynamics and neuronal activity in sleep- and wake-promoting areas remains largely unknown. Although many brain areas involved in sleep control have been identified [9, 10], the complex nonlinear dynamics of extended neuronal networks (comprised of diverse mutually connected neuronal populations) continues to pose severe limitations to a mechanistic understanding of the collective neuronal behavior underlying observed sleep-stage transition patterns. This is a general problem that arises in systems where the emerging behavior at the system level originates from collective dynamics of a large number of interacting units. While elegant theories bridging micro- and macro-scales are sometimes available for such systems at thermodynamic equilibrium, emergent collective phenomena out of equilibrium often require a top-down approach, where basic mechanisms are inferred from the statistical characteristics of emergent dynamics.

Following this approach, here we investigate the temporal organization in bursting activity of brain rhythms across the sleep-wake cycle, with the aim to understand the basic physical principles bridging neuronal interactions at the microscopic level and physiologic state at the system level. The present study is motivated by recent works showing that the complex dynamics of sleep stage transitions give rise to power-law probability distributions for the durations of brief awakenings and arousals, a robust scale-invariant organization observed in both humans and animal models [13, 26–31]. Power-law distributions *P*(*x*) = *Nx*^−*α*^, where *α* is the scaling exponent and *N* is a normalization constant, are the statistical hallmark of scale invariance, i.e., they are not altered by a change of scale from *x* to *Lx*, hence lacking a relevant characteristic scale. Power laws are typical features of physical systems at the critical point of a second order phase transition in equilibrium thermodynamics [32, 33]. At criticality systems exhibit high susceptibility and sensitivity to interactions among elements, leading to emergent collective behavior across scales, and thus, power laws. The critical point is located at the border between an ordered and a disordered phase, and can be reached by fine tuning external parameters. In contrast to this scenario, in non-equilibrium systems the dynamics can be spontaneously driven at criticality, where an active phase characterized by bursts/avalanches with power-law distributed sizes and durations coexists with a quiescent phase with exponential-like statistics [15, 17, 34, 35]. We note that such avalanche criticality does not necessarily co-occur with edge-of-chaos criticality [36].

Since brief awakenings/arousals can be viewed as ‘active’ states of the brain that interrupt the ‘inactive’ phase represented by sleep periods, the scale-invariant organization of arousals has been interpreted as a fingerprint of criticality in sleep dynamics [13, 26, 31, 37]. Contrary to the established interpretation of arousals as random and detrimental disruptions of sleep [38, 39], caused by external stimuli or as part of the patho-physiology of sleep disorders [12, 14, 40–44], such robust scale-invariant temporal organization indicates that arousals are an integral part of sleep regulation, resulting from a single dynamic principle responsible for the emergent temporal organization of sleep stages and sleep-stage transitions.

The hypothesis that sleep phenomenology and micro-architecture may reflect underlying scale-invariant dynamics resulting from self-tuning of a system at criticality [34] would imply presence of power-law distributions and long-range correlations as basic characteristics for sleep-related neuronal activity across spatial and temporal scales. Although power-law distributions [45–47] and long-range correlations [48] have been previously reported in neuronal and brain dynamics, and some attempts to correlate them with behavioral scaling laws has been made [49], the connection between (i) dynamics of sleep-related brain waves activity, (ii) neuronal circuitry and pathways related to sleep regulation, and (iii) emergent scale-invariant organization of brief arousals/awakenings, remains not understood.

To test this hypothesis, we consider the dominant brain waves in the sleep-wake cycle of rats, and study their dynamics and coupling in relation to the neuronal circuitry responsible for wake and sleep control. We focus on sleep-promoting PZ [50], a region of the rostral medulla, that plays a significant role in the regulation of slow-wave sleep [6–8, 51]. Both PZ cell body specific lesion and disruption of GABAergic transmission in PZ result in insomnia, indicating that within the PZ, GABAergic neurons are primarily in sleep control. Similar to other NREM sleep promoting neurons, PZ GABAergic neurons project to and inhibit wake promoting systems, such as the parabrachial neurons that project to basal forebrain neurons, which in turn project to the cortex and are critical for cortical activation and wakefulness [6]. It has been shown that lesions of all PZ neurons result in a significant decrease of total NREM sleep during the light period (when rats are predominantly asleep), whereas chemogenetic activation of PZ neurons results in an increase of NREM sleep and slow-wave activity with dominant *δ* rhythm [6]. However, the influence of PZ sleep-promoting neurons on cortical brain waves activity remains not clear. In particular, the role played by PZ neurons in the dynamics and temporal organization of *δ* waves, as well as in the emergence of transient *θ*-bursts arousal activations during the sleep-wake cycle, has not been investigated.

To this end, we analyze long-term continuous EEG recordings in control rats and rats where the PZ brain area is lesioned, and we investigate the complex dynamics of *θ*- and *δ*-bursts in relation to PZ neuronal integrity, during both light and dark periods. In particular, we focus on the emergent scale-invariant features in the temporal organization of *θ*- and *δ*-bursts, and study whether alterations in the sleep-wake cycle are mirrored by a reorganization of dominant brain rhythms, a question that has not been addressed so far in sleep research. Our aim is to establish basic mechanisms that underlie cortical dynamics and sleep micro-architecture, and whether these dynamics exhibit characteristics of a system at criticality. Confirming our hypothesis, that cortical activations underlying sleep micro-architecture exhibit critical dynamics, would lay the foundation for a novel unified framework of the sleep-wake cycle, where sleep and arousals/wake, consolidated and transient states, originate from the same fundamental principles and a common mechanism that bridge collective behaviors across spatio-temporal scales, from neuronal assemblies to brain rhythms and emerging physiologic states.

## Results

### Transient dynamics in bursting activity of *θ* and *δ* rhythms

**Fig 1:**
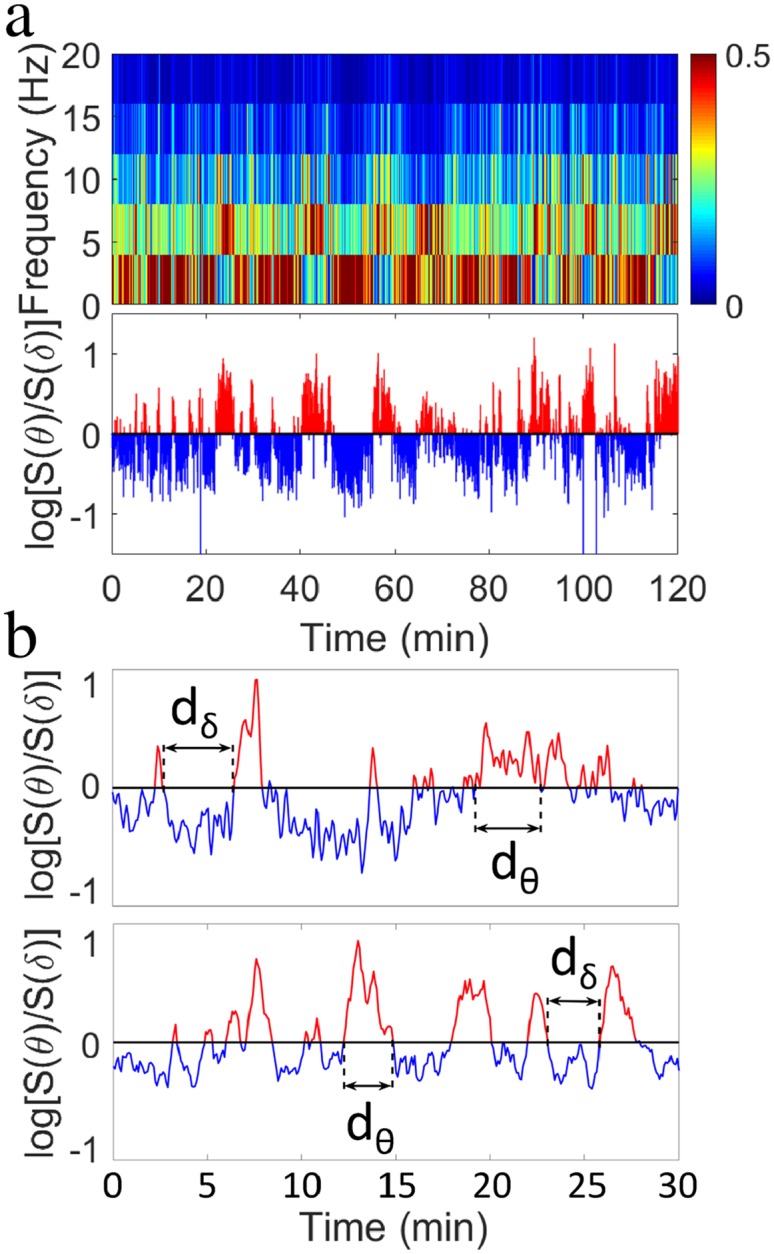
Cortical activity across the sleep-wake cycle is characterized by intermittent irregular transitions between brain rhythms with dominant spectral power. (a) (Top panel) Spectrogram obtained from cortical EEG signal of a control group rat over a 2 h segment of 12-hour lights-on period (when rats predominantly sleep). Spectral power is calculated in non-overlapping time windows *w* = 5 s, and is color coded over a range (0-20 Hz) of physiologically-relevant frequencies. Segments in red indicate bursts of prominent activity in the low frequency band (0-4 Hz, corresponding to *δ* waves) and intermediate frequency band (4-8 Hz, corresponding to *θ* waves). (Bottom panel) Ratio *R*_*θδ*_ = *S*(*θ*)/*S*(*δ*) of the spectral power in the *θ* and *δ* band in logarithmic scale obtained for each window *w* from the spectrogram shown in top panel. Values *R*_*θδ*_ above a threshold *Th* = 0 indicate predominance of *θ* rhythm (in red), while values below the threshold *Th* = 0 correspond to predominance of *δ* rhythm (in blue). (b) Smoothed ratio *R*_*θδ*_ of the spectral power in the *θ* and *δ* band during a 30 min segment of 12-hour dark (lights-off) period for a control rat (top panel) and a PZ-lesioned rat (bottom panel). *R*_*θδ*_ is calculated in non-overlapping windows *w* = 5 s; smoothing is performed using a 5 point moving average. *θ*- and *δ*-bursts are defined as sequences of consecutive windows *w* where either the power in *θ* or *δ* band is dominant.

To dissect the temporal organization of *δ*- and *θ*-bursts in the broadband brain activity during sleep and wake, we analyzed the time course of the EEG signal by evaluating the spectral power in several frequency bands on non-overlapping windows of length *w* (Materials and methods, Data analysis). In Fig 1a we show a typical spectrogram *S*(*f*) as a function of time for a 2 h recording of a rat in the control group. We notice that, in each window, the spectral power is primarily concentrated in either the *δ*-wave frequency range (0 − 4Hz) or in the *θ*-wave band (4 − 8Hz), and we observe sharp transitions from periods with dominant *δ* to periods with dominant *θ* waves. Such dynamics can be understood as the temporal evolution of the ratio *R*_*θδ*_ = *S*(*θ*)/*S*(*δ*) between *θ* and *δ* spectral power in association with different physiological states—NREM, REM and arousals/wake (Fig 1 shows the transient dynamics of bursts in *δ*- and *θ*-waves power represented by the logarithm of *R*_*θδ*_ as a function of time *t*).

The ratio *R*_*θδ*_(*t*) exhibits irregular, intermittent fluctuations between values larger and smaller than a threshold *Th*—a typical characteristic of non-equilibrium dynamics: *R*_*θδ*_ > *Th* = 1 indicates that the spectral power in the *θ*-wave band is dominant; vice-versa, for *R*_*θδ*_ < *Th* = 1 the spectral power is dominated by the *δ*-wave. We define bursts in *θ* and *δ* brain rhythms as sequences of consecutive time windows where *R*_*θδ*_ > *Th* = 1 and *R*_*θδ*_ < *Th* = 1, respectively (Fig 1b). The focus of this study is to establish the dynamical features and underlying mechanisms of bursts in cortical activity across the sleep-wake cycle by investigating the temporal organization of such bursts and their coupling. To this aim, we associate a duration *d* = *n* * *w* to each burst (Materials and methods, Data analysis), where *n* is the number of consecutive windows belonging to a given burst and *w* is the window length (Fig 1b).

### Distinct functional forms of *θ*- and *δ*-burst duration distributions

**Fig 2:**
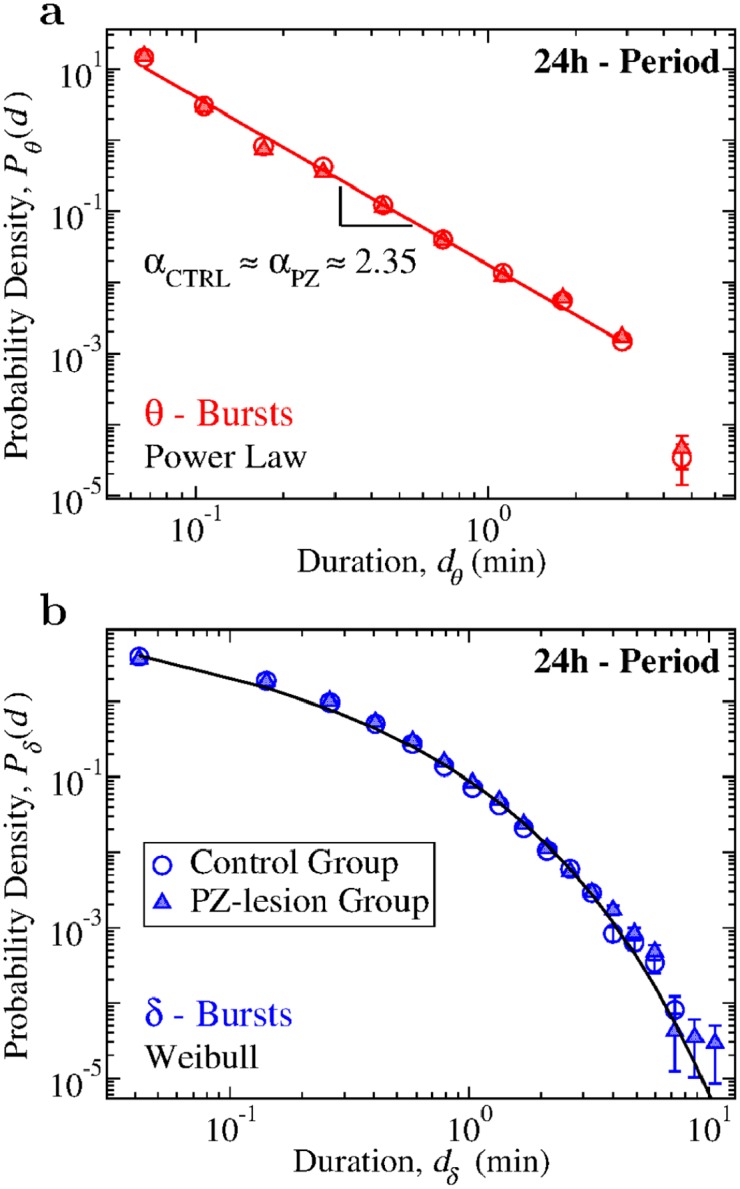
Durations of *θ*- and *δ*-bursts across the 24 h sleep-wake cycle follow distinct statistics that are robust, do not change with lesion of the PZ neurons, and are typical for non-equilibrium systems at criticality. (a) Probability density distribution of *θ*-burst durations for control (open circles) and PZ-lesioned rats (full triangles) over the 24 h period (pooled data). The distribution exhibits a power-law behavior in both groups (red tick line), with exponent *α*_*ctrl*_ = 2.34 ± 0.06 for control rats and *α*_*PZ*_ = 2.31 ± 0.07 for PZ. The power-law exponent value for control and PZ rats do not show significant difference (*t*-test, *p* > 0.99). Lesion of the PZ area does not cause significant variations in the power-law exponent *α*, indicating a robust scale-invariant temporal organization of *θ*-bursts. (b) Probability density of *δ*-burst durations for control and PZ-lesioned rats over 24 h period (pooled data). In contrast to the statistics of *θ*-bursts, *δ*-bursts durations follow a Weibull distribution (stretched exponential tail with a characteristic time scale, Eq 2). Parameters of the Weibull functional form are not significantly affected by lesion of the PZ (*β*_*ctrl*_ = 0.59, λ_*ctrl*_ = 0.16; *β*_*PZ*_ = 0.54, λ_*PZ*_ = 0.13). Black line is a Weibull fit of the distribution for control rats. All durations are calculated using window size *w* = 5 s for the spectrogram and threshold *Th* = 1 on the ratio *R*_*θδ*_ (Fig 1). Error bars are calculated for each value and where not shown are smaller than the symbol size. Error bars calculation and binning procedure are described in Materials and methods, Data analysis.

We next study the probability distribution of the durations of *θ*- and *δ*-bursts over a 24h period for control and PZ-lesioned rats (Materials and methods, experimental setup). We notice that *θ* and *δ* bursts follow very different statistics. The probability density *P*_*θ*_ of the *θ*-burst durations exhibits a power-law behavior, followed by an exponential cut-off (Fig 2a),

$$\begin{array}{c} P_{\theta }\left(d\right)∼d^{-\alpha }, \end{array}$$ (1)

where *α* denotes the scaling exponent of the power law. Power laws are the fingerprint of scale invariance and, depending on the context, they imply that events of any size, length or duration are likely to occur with some finite probability that is larger than expected in random or short-range correlated processes. Presence of power law indicates absence of characteristic time scales in the underlying control mechanism, which is a typical feature of physical systems at the critical point of continuous phase transition—a highly sensitive state where cooperative behavior spontaneously emerges over a range of time scales characterized by long-range correlations (presence of such correlations in the duration of consecutive *δ*- and *θ*-bursts is shown in Fig 7 and is discussed later in the manuscript). Notably, such scale-invariant power-law behavior is not influenced by lesions of the PZ neurons: in both control and PZ-lesioned group we find an exponent of *α* ≃ 2.35 (Fig 2a), indicating that lesions of the PZ do not alter the dynamical micro-architecture of *θ*-bursts across the 24h sleep-wake cycle.

In contrast to the power-law characteristic of *θ*-bursts, *δ*-burst durations follow a distinct behavior that is described by a Weibull distribution (Fig 2b),

$$\begin{array}{c} P_{\delta }\left(d;\beta ,\lambda \right)=\frac{\beta }{\lambda }{\left(\frac{d}{\lambda }\right)}^{\beta -1}e^{-{\left(d/\lambda \right)}^{\beta }}, \end{array}$$ (2)

where λ is the characteristic time scale and *β* is the shape parameter. Further, we find that the distribution of *δ*-burst durations follows the same Weibull functional form for both control and PZ-lesioned groups, with similar values of the Weibull parameters λ and *β*.

The functional forms established for the distributions of *θ*- and *δ*-burst durations in Eqs 1 and 2 and Fig 2, indicate a very different temporal organization of *θ*- and *δ*-bursts. A surrogate test based on randomizing the sequence of windows *w* in the EEG spectrogram (Fig 1a) leads to a different profile of *θ*- and *δ*-burst durations with an exponential functional form for their distributions (Fig A in S1 File), indicating that the observed temporal organization in bursting activity of brain rhythms is physiologically relevant and relates to underlying regulation.

The results demonstrate a remarkable duality of power-law scale-invariant dynamics for *θ*-bursts and Weibull dynamics with characteristic time scale for *δ*-bursts. Such duality of two unlikely processes appears to be a general feature of brain cortical activity of individual subjects in each group across the entire sleep-wake cycle. Moreover, this feature is robust as it remains unchanged in both control and PZ-lesioned rats. Coexistence of scale-invariant and exponential type behaviors is a hallmark of non-equilibrium systems at criticality characterized by alternating active and inactive states, and exhibiting self-organization (i.e., maintaining critical behavior without external tuning) [35, 52, 53]. Thus, our observations indicate an intrinsic common mechanism that underlies the temporal organization of bursting activity in both *δ* and *θ* cortical waves across the distinct physiological states of wake/arousals and sleep.

**Fig 3:**
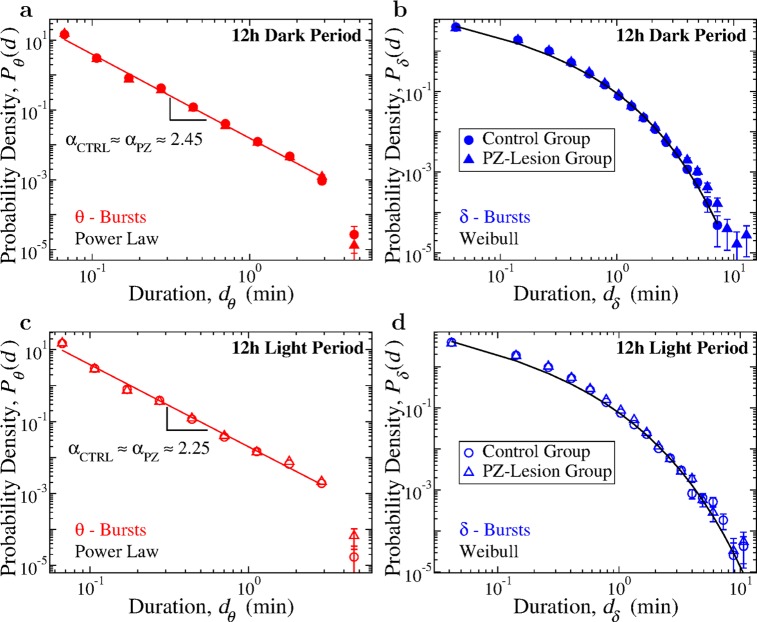
Critical behavior represented by duality of power-law and Weibull distribution for *θ*- and *δ*-bursts characterizes cortical activity during both dark and light periods in control and PZ-lesioned rats. (a) Probability distributions of *θ*-burst durations for control (circles) and PZ-lesioned (triangles) rats over the 12 h dark lights-off period (pooled data) follow a power-law with an exponent *α*_*ctrl*_ = 2.44 ± 0.06 and *α*_*PZ*_ = 2.40 ± 0.06 (higher than for the 24 h sleep-wake cycle, Fig 2), where the line shows a power-law fit for the control group. (b) Probability distributions of *δ*-burst durations for control and PZ-lesioned rats over 12 h dark period (pooled data) follow a Weibull form, with no significant differences in the fitting parameters (*β*_*ctrl*_ = 0.59, λ_*ctrl*_ = 0.16; *β*_*PZ*_ = 0.54, λ_*PZ*_ = 0.14), where the line shows a Weibull fit for the control group. (c) Probability distributions of *θ*-burst durations for control and PZ-lesioned rats over the 12h lights-on period (pooled data) also follow a power-law but with smaller exponent *α*_*ctrl*_ = 2.28 ± 0.07 and *α*_*PZ*_ = 2.24 ± 0.08 compared to the dark period, indicating higher probability for longer durations. (d) Probability distributions of *δ*-burst durations for control and PZ-lesioned rats over the 12 h light period (pooled data) follow Weibull behavior for both groups with no significant differences in the fitting parameters (*β*_*ctrl*_ = 0.54, λ_*ctrl*_ = 0.12; *β*_*PZ*_ = 0.56, λ_*PZ*_ = 0.14). Lines in (c) and (d) show fits for the distributions of the control group. All durations are calculated using window size *w* = 5 s for the spectrogram and threshold *Th* = 1 on the ratio *R*_*θδ*_ (Fig 1). Error bars are calculated for each value and where not shown are smaller than the symbol size. Error bars calculation and binning procedure are described in Materials and methods, Data analysis. The power-law exponent value for control and PZ rats do not show significant difference for both light and dark periods (*t*-test, *p* > 0.46).

To achieve a more detailed understanding of the temporal organization of bursting activity of *θ*- and *δ*-waves during sleep and wake, and the role of PZ neurons in these dynamics, we next analyze the probability distributions of *θ*- and *δ*-burst durations separately during 12-hour dark (nighttime) and light (daytime) periods. In contrast to humans, rats are predominantly awake during the dark period and asleep during the light period. Although sleep and wake are characterized by different dominant brain rhythms with distinct dynamics, synchronization and coupling patterns across cortical areas [25, 54], our analyses show that the duality of power-law for the *θ*-bursts and Weibull distribution for the *δ*-bursts durations is robust and remains independent of the dominant physiologic state (sleep or wake). Such coexistence of scale-invariant and scale-specific temporal organization in *θ*- and *δ*-bursts appears to be a basic characteristic of cortical activity during both dark and light periods (Fig 3). Importantly, the scaling exponent *α* of the power law as well as the Weibull parameters λ and *β* concurrently change comparing dark to light periods, indicating a coordinated modulation of *θ* and *δ* brain dynamics across sleep and wake. This rises the hypothesis of a coupling mechanism controlling the durations of consecutive *θ*- and *δ*-bursts (confirmed by empirical results shown in Fig 8). The observations are consistent for both control and PZ-lesioned group, demonstrating a remarkable robustness of the power-law and Weibull functional form, which remain present across the dark and light periods, and even after lesioning the PZ neurons (Fig 3).

Further, considering separately dark and light periods, we observe that the power-law scaling exponent *α* is higher for the dark period (Fig 3a and 3c), indicating that *θ*-bursts of longer duration are more likely during the light period when rats are predominantly asleep. Higher probability for longer lasting *θ*-bursts during light periods could be associated with the presence of longer episodes of REM sleep, where *θ*-wave oscillations are dominant. This leads to an average increase of spectral power in the *θ* band, and thus, to a higher likelihood for longer *θ*-bursts (smaller *α*) during the light period. For both control and PZ-lesioned rats, the scaling exponent *α* ≈ 2.45 is higher during the dark period, and lower *α* ≈ 2.25 during the light period (Fig 3a and 3c), in concurrence with change in Weibull parameters λ and *β* (Fig 3b and 3d). Notably, comparing the control vs the PZ-lesioned group within a given dark or light period, we find no significant difference for the distributions of *θ*- and *δ*-burst durations, indicating that the temporal organization of these fundamental brain rhythms across the sleep-wake cycle is not influenced by neuronal assemblies in the PZ area.

### Robust organization of *θ*- and *δ*-bursts across time scales

Our findings show that bursts associated with *θ* and *δ* rhythms exhibit a distinct temporal organization described by specific duration distributions: power law for *θ*-bursts indicating absence of a characteristic time scale (i.e. scale-invariant behavior), and Weibull distribution for *δ*-bursts with a characteristic time scale λ (Figs 2 and 3). These findings are obtained based on a particular choice for the observational window size *w* and threshold *Th* utilized to analyze bursting dynamics (Fig 1; Materials and methods, Data analysis). We note that in our analyses, *θ*- and *δ*-bursts are defined as time periods for which the ratio *R*_*θδ*_ = *S*(*θ*)/*S*(*δ*) is above or below the threshold *Th* = 1, where *R*_*θδ*_ is evaluated over consecutive windows of *w* = 5 s. To demonstrate that our results are indeed independent of the particular choice of parameters *Th* and *w*, we repeat the analyses for a range of parameter values. We find that universal scaling functions describe the dynamics of burst durations across the 24-hour sleep-wake cycle.

**Fig 4:**
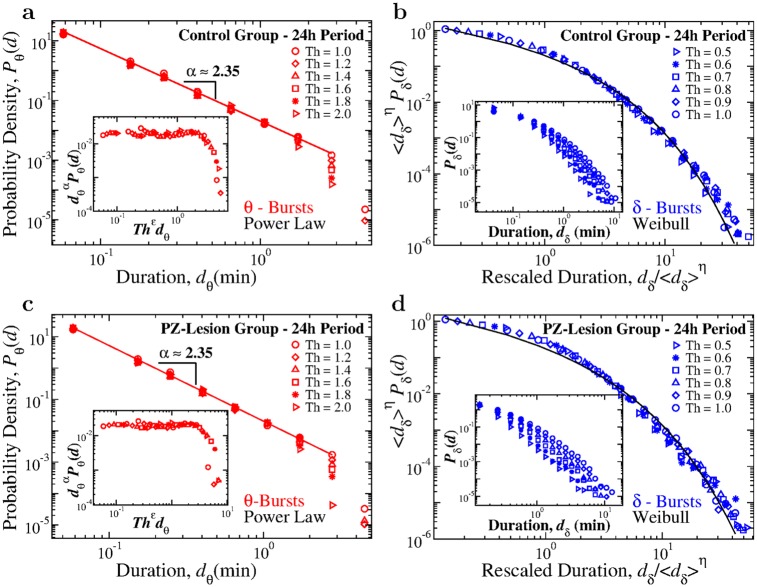
Critical characteristics in temporal dynamics of bursts in dominant rhythms are a fundamental feature of cortical activity across the sleep-wake cycle independent of thresholds utilized to define bursts. The distribution functional forms of power-law for *θ*-bursts durations and Weibull for *δ*-bursts durations remain preserved for different threshold values *Th* imposed on the ratio *R*_*θδ*_ (Fig 1). (a) Distributions of *θ*-burst durations for control rats over a 24h period (pooled data) evaluated using different *Th* values consistently follow the same power-law behavior (red line), with an exponential cut-off that is controlled by *Th*. With increasing *Th* the distribution cut-off shifts towards shorter burst durations, a finite size effect typically observed in systems at criticality. Inset: data for different *Th* collapse onto a single universal function *f*_*θ*_ when we plot *P*(*d*)*d*^*α*^ versus *Th*^*ϵ*^*d*, with *α* = 2.35 and *ϵ* = 0.8. (b) Rescaled distributions of *δ*-burst durations for control rats over a 24 h period (pooled data) obtained for different *Th* values, consistently follow the same Weibull form. Distributions are rescaled by 〈*d*_*δ*_〉^*η*^, where 〈*d*_*δ*_〉 is the mean *δ*-burst duration and *η* = 1.2. After rescaling, distributions collapse onto a single function following a Weibull behavior, *f*(*d*; λ, *β*) (black line) with λ = 0.55 and *β* = 0.59. Inset: Distributions *P*_*δ*_ for different thresholds *Th* (not rescaled). (c) Distributions of *θ*-burst durations for PZ-lesioned rats over a 24 h period (pooled data) evaluated using different threshold values *Th* consistently follow the same power-law behavior (red line), with an exponential cut-off controlled by *Th*. Inset: Data collapse onto a universal function *f*_*θ*_ by plotting *P*(*d*)*d*^*α*^ versus *Th*^*ϵ*^*d* with *α* = 2.35 and *ϵ* = 0.8 (same as for control rats in a). (d) Rescaled distribution of *δ*-burst durations for PZ-lesioned rats over a 24 h period (pooled data) obtained for different *Th* values follow the Weibull form. Distributions are rescaled by 〈*d*_*δ*_〉^*η*^, with 〈*d*_*δ*_〉 mean *δ*-burst duration and *η* = 1.2. After rescaling, the distributions collapse onto a single Weibull distribution *f*(*d*; λ, *β*) (black line) with λ = 0.44 and *β* = 0.54. Inset: Distributions *P*_*δ*_ for different thresholds *Th* (not rescaled). Results in all panels are obtained for a fixed scale of analysis, keeping the window size *w* = 5 s (Fig 1). Results are consistent when considering separately light and dark periods (Fig B and Fig C in S1 File).

We first examine the duration distributions of *θ*- and *δ*-bursts for different threshold values *Th*, keeping the window size *w* fixed. By increasing the threshold on the ratio *R*_*θδ*_ from *Th* = 1 to *Th* = 2, we find that the scaling exponent *α* characterizing the power-law distribution of *θ*-burst durations remains stable (data collapse on to a single curve, Fig 4a and 4c). The scaling behavior is followed by an exponential cut-off that, with increasing *Th* values, shifts to shorter burst durations *d*_*θ*_.

For both control and PZ-lesioned rats this behavior is captured by the relation

$$\begin{array}{c} P_{\theta }\left(d_{\theta }\right)∼d_{\theta }^{-\alpha }f_{\theta }(\frac{d_{\theta }}{Th^{-\epsilon }}), \end{array}$$ (3)

where *f*_*θ*_(*d*_*θ*_/*Th*^−*ϵ*^) is a universal scaling function, *α* is the power-law scaling exponent and *ϵ* expresses the dependence of the cut-off on *Th*. This universal scaling function is confirmed by the data collapse obtained by plotting $d_{\theta }^{\alpha }P_{\theta }\left(d\right)$ versus *d*_*θ*_/*Th*^−*ϵ*^ for a range of *Th* values (insets in Fig 4a and 4c). In addition to the universal scaling function, such exponential cut-off is reminiscent of finite size effects observed in systems at criticality.

Similarly, we demonstrate that a unique function *f*_*δ*_ describes the distribution of *δ*-burst durations that is independent of the threshold *Th* (Fig 4b and 4d). Since a *δ*-burst is defined as a time period of consecutive windows *w* where *R*_*θδ*_ < *Th* = 1 (Fig 1b), to properly explore the behavior of the duration distribution for states with increasingly dominant *δ* power, we repeat the analyses for different values *Th* < 1. We observe that as *Th* decreases the probability for long *δ*-bursts decreases, while short *δ*-bursts become more likely (insets in Fig 4b and 4d). However, when distributions are rescaled by their respective mean *δ*-burst duration 〈*d*_*δ*_〉, they all collapse onto a unique function *f*_*δ*_, which is the same for both control and PZ-lesioned rats (shown in main panels of Fig 4b and 4d respectively). This universal function *f*_*δ*_ is defined by the scaling relation,

$$\begin{array}{c} P_{\delta }\left(d_{\delta }\right){∼}_{\delta }\rangle^{-\eta }f_{\delta }{\left(d_{\delta }{/}_{\delta }\right\rangle }^{\eta }), \end{array}$$ (4)

where *η* = 1.2 for both rat groups, and is well fitted by a Weibull functional form as we find by rescaling *d*_*δ*_ and *P*_*δ*_.

Repeating the analyses for 12-hour dark and light periods separately, we find that the universal scaling forms in Eqs 3 and 4 consistently describe the dynamics of *δ*- and *θ*-bursts in both control and PZ-lesioned groups (Fig B and Fig C in S1 File). Thus, our results indicate that the duality of power-law and Weibull distribution, as well as the scaling properties summarized in Eqs 3 and 4, are robust features of the bursting dynamics of *θ* and *δ* rhythms across the sleep-wake cycle, and do not depend on the particular threshold *Th* utilized to study the time evolution and intermittent dynamics of *θ*- and *δ*-bursts embedded in the EEG spectral power.

**Fig 5:**
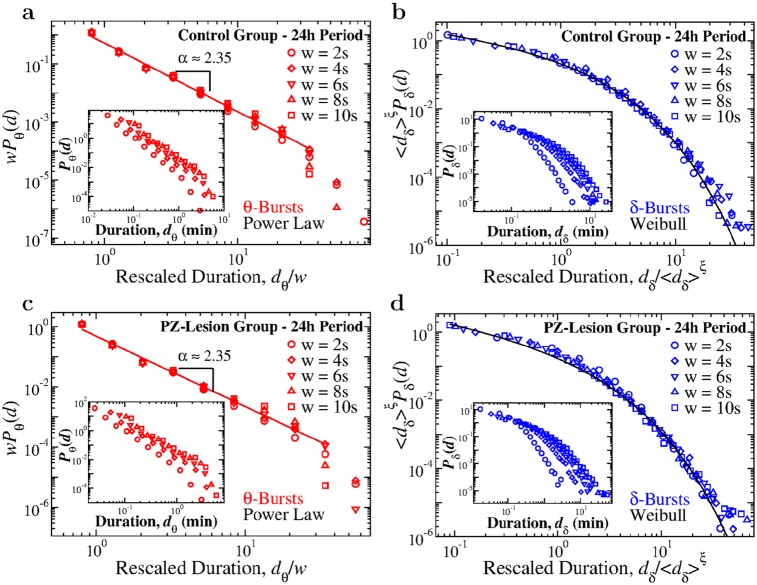
Critical characteristics in temporal dynamics of bursts in dominant cortical rhythms are independent of the scale of analysis. Distributions of *θ*- and *δ*-burst durations for different scales of observation defined by the window size *w* (Fig 1). (a) Rescaled distribution *P*_*θ*_ of *θ*-burst durations for control rats over a 24 h period (pooled data). Distributions obtained for different scales of observation are rescaled by the window size *w* and consistently show the same power-law behavior with *α* = 2.35, as proven by the data collapse (red line). Small deviations observed on the tail of *P*_*θ*_ for *w* > 6 are due to coarse-graining effect at large-window sizes. Inset: Distributions *P*_*θ*_ for different window sizes *w* (not rescaled). (b) Rescaled distribution of *δ*-burst durations for control rats over a 24 h period (pooled data). Distributions obtained for different window sizes *w* are rescaled by 〈*d*_*δ*_〉^*ξ*^, where 〈*d*_*δ*_〉 is the mean *δ*-burst duration and *ξ* = 1.2, and collapse onto a single function that is well described by a Weibull distribution *f*(*d*; λ, *β*) with λ = 0.55 and *β* = 0.59 (black line). Inset: Distributions *P*_*δ*_ for different window sizes (not rescaled). (c) Rescaled distribution of *θ*-burst durations for PZ-lesioned rats over a 24h period (pooled data). Distributions obtained for different window sizes *w* are rescaled by the corresponding window size, and consistently show the same power-law behavior with *α* = 2.35, as proven by the data collapse (red line). Small deviation observed on the tail of *P*_*θ*_ for *w* > 6 are due to large-window effects. Inset: Distributions *P*_*θ*_ for different window sizes (not rescaled). (d) Rescaled distribution of *δ*-burst durations for PZ-lesioned rats over a 24 h period (pooled data). Distributions are rescaled by 〈*d*_*δ*_〉^*ξ*^, with *ξ* = 1.2, and collapse onto a single function following a Weibull behavior *f*(*d*; λ, *β*) (black line) with λ = 0.44 and *β* = 0.54. Inset: Distributions *P*_*δ*_ for different window sizes (not rescaled). Results in all panels are obtained for fixed threshold *Th* = 1 on the ratio *R*_*θδ*_ (Fig 1). Results are consistent when considering separately light and dark periods (Fig D and Fig E in S1 File).

Moreover, we also find that the functional behavior of the distributions of *θ*- and *δ*-burst durations is to a large extend independent of the window size *w*, used to investigate the time course of the EEG spectral power (Fig 1a). Intuitively, larger *w* would tend to fail in identifying short bursts and merge them together, thus causing an increase in the probability of observing longer durations. In that regard, considering the power-law temporal organization of *θ*-bursts, larger window sizes *w* mainly influence the tail of the distribution with longer *θ*-bursts durations, thus leading to decrease of the scaling exponent *α* (insets in Fig 5a and 5c). We note that the window size effect becomes visible only for extremely large *w* compared to the average *θ*-burst duration 〈*d*_*θ*_〉. However, when the *θ*-bursts distributions (curves in insets of Fig 5a and 5c) are rescaled by their respective window size *w*, all data collapse onto a single power law (Fig 5a and 5c), confirming the robustness of the results obtained in Fig 2a for both control and PZ-lesioned rats. This rescaling is represented by the following relation

$$\begin{array}{c} P_{\theta }\left(d\right)∼w^{-1}\cdot f_{\theta }\left(d/w\right). \end{array}$$ (5)

Separate analyses of 12-hour dark and light periods for different window sizes *w* (shown in Fig D a and c, Fig E a and c in S1 File) further confirm the robustness of the established scale-invariant power-law form for the *θ*-burst durations (Fig 3a and 3c).

A similar data collapse characterizes the dependence of the *δ*-burst duration distribution on window size *w*. Generally, we observe that for increasing *w* the probability for long *δ*-bursts increases, while short *δ*-bursts become less likely (insets in Fig 5b and 5d). We find that the relation between *w* and the average duration of *δ*-bursts 〈*d*_*δ*_〉 is given by *w* ∼ 〈*d*_*δ*_〉^*ξ*^, with exponent *ξ* = 1.2 for both control and PZ-lesioned rats. When *δ*-burst duration distributions corresponding to different window sizes *w*, are rescaled by their respective mean duration 〈*d*_*δ*_〉, we find that all distributions collapse onto a unique function *f*_*δ*_ of a Weibull form (Fig 5b and 5d), expressed by the following scaling relation

$$\begin{array}{c} P_{\delta }\left(d\right)∼w^{-1}\cdot f_{\delta }\left(d/w\right)∼{\left\langle d_{\delta }\right\rangle }^{-\xi }\cdot f_{\delta }\left(d/{\left\langle d_{\delta }\right\rangle }^{\xi }\right). \end{array}$$ (6)

As in the case for *θ*-burst dynamics, we find that the temporal organization of *δ*-bursts is robust and characterized by a scaling function (Eq 6), which is universal for the control and PZ-lesioned groups, and remains stable during light and dark periods (Fig D b and d, Fig E b and d in S1 File).

The existence of universal scaling functions (Eqs 3–6) not only demonstrates that duration distributions are independent of the specific set of parameters used to identify *θ*- and *δ*-bursts, but also constitutes a striking evidence of scale invariance, a property associated with systems operating at criticality.

### Self-similar micro-architecture of active and quiet states in the sleep-wake cycle

Our investigations reveal a scale-invariant power-law structure for the durations of *θ*-bursts and a homogeneous functional form of Weibull type with a characteristic time scale for *δ*-burst durations, that are remarkably robust during light and dark periods across the sleep-wake cycle, and are consistent for both the control and PZ-lesioned group (Figs 2 and 3). The coexistence of these two distinct types of dynamics draws a strong parallel with far-from-equilibrium physical phenomena that are characterized by bursting dynamics and abrupt transitions between active and quiet states, such as avalanches and earthquakes [15, 35, 55–57]. For instance, the energy released during avalanches/earthquakes (active states) is also distributed according to a power law, while the distribution of time intervals between consecutive avalanches/earthquakes (quiet states) is described by a generalized Gamma distribution with a characteristic time scale (exponential tail). Gamma is a universal scaling function that is independent of spatial scales and minimum magnitude thresholds, and is consistently observed for a broad range of conditions despite the large variability associated with phenomena such as earthquakes and avalanches [55, 58–60].

**Fig 6:**
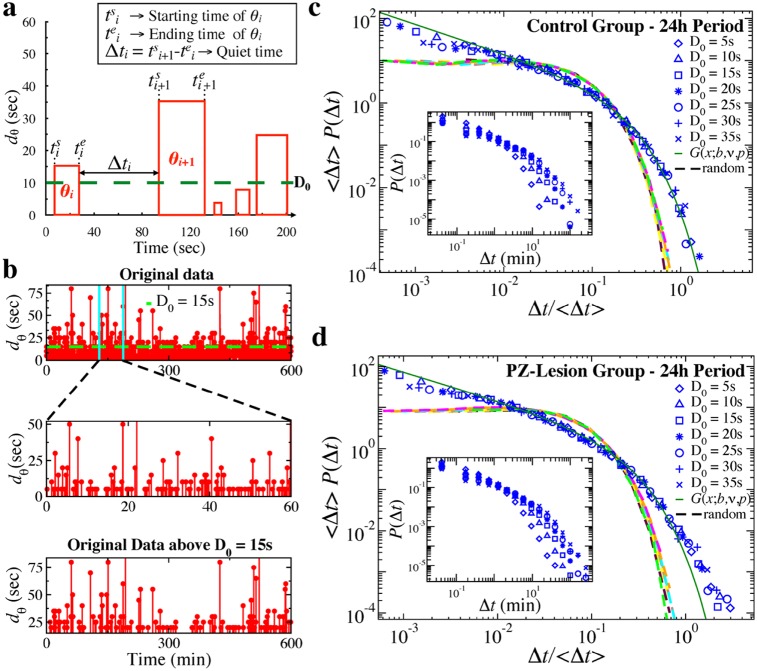
Self-similar structure in quiet times between consecutive *θ*-bursts indicates coupling between time of occurrence and burst duration. (a) Schematic diagram of quiet time Δ*t* between consecutive *θ*-bursts. A quiet time Δ*t*_*i*_ is the time elapsed from the end of burst *θ*_*i*_ to the beginning of the following burst *θ*_*i*+1_. (b) Top: Time series of *θ*-burst durations for about 600 min recording of a control rat. Middle: A 60 min segment from the sequence shown in top panel. Bottom: Sequence comprised only of the *θ*-burst durations longer than *D*_0_ = 15 s that are present in the 600 min time series shown in the top panel. Selecting only bursts longer than *D*_0_ = 15 s, the temporal pattern at the scale of 600 min looks similar to pattern at smaller scale of 60 min, indicating self-similar structure in quiet times. (c) Distribution of quiet times for different thresholds *D*_0_ on *θ*-burst durations over a 24 h period in control rats (blue symbols). When rescaled by 〈Δ*t*〉 (main panel), distributions obtained for different *D*_0_ collapse onto a unique function that is well described by a generalized Gamma distribution *G*(*x*; *b*, *ν*, *p*) (solid green line), with *b* = 0.15, *ν* = 0.31, and *p* = 0.91. Applying the same procedure to a sequence of randomly reshuffled *θ*-burst durations, thus eliminating information about the timing of *θ*-bursts, leads to distributions that collapse onto an exponential function (dashed lines). Inset: Distributions of quiet times for different thresholds *D*_0_ before rescaling. (d) Distributions of quiet times for different thresholds *D*_0_ on *θ*-burst durations over a 24 h period in PZ-lesioned rats. Distributions collapse onto a unique function when rescaled by 〈Δ*t*〉 (main panel). Similar to control rats, this function is well described by a generalized Gamma function *G*(*x*; *b*, *ν*, *p*) (solid green line), with *b* = 0.17, *ν* = 0.24, and *p* = 0.83. Distribution of quiet times obtained from a sequence of randomly reshuffled *θ*-burst durations collapse onto an exponential distribution (dashed lines). Insets: Distributions of quiet times in PZ-lesioned rats for different thresholds *D*_0_ before rescaling. Results are consistent when considering separately light and dark periods (Fig F in S1 File).

In the context of sleep dynamics, wake and brief arousals during sleep can be considered as active states that, in rodents, are characterized by bursts in *θ* rhythms. Thus, we focus on the organization of *θ* rhythms in time. Specifically, we investigate the relationship between the duration of *θ*-bursts and their occurrence in time (Fig 6), and we hypothesize that a self-similar structure, invariant across time scales, may also characterize the occurrence of *θ*-bursts. In analogy with non-equilibrium phenomena, where quiet times are associated with the magnitude of active states, presence of a self-similar structure between occurrence and duration of *θ*-bursts (active states) would provide additional evidence for criticality in sleep micro-architecture.

To this end, we consider the time sequence of *θ*-bursts, and we investigate the statistical features of the quiet times Δ*t* separating consecutive bursts, taking into account the duration *d*_*θ*_ of each *θ*-burst (Fig 6a). Since *θ*-bursts vary in duration, we impose a threshold *D*_0_ representing the time scale of analysis, and we define quiet time Δ*t*_*i*_ as the period from the end of *θ*_*i*_-burst to the beginning *θ*_*i*+1_-burst. Thus, the statistical characteristics of Δ*t*_*i*_ depend on the threshold value *D*_0_. We obtain the probability distribution *P*(Δ*t*; *D*_0_) of quiet times Δ*t*_*i*_ for different values of *D*_0_ (insets in Fig 6c and 6d). With increasing threshold (scale of observation) *D*_0_, the probability of longer Δ*t*_*i*_ increases, while the probability of short quiet times decreases, leading to different curves for the distributions *P*(Δ*t*; *D*_0_).

Visual inspection of the complex profile formed by the time sequence of *θ*-bursts and their respective durations shows an apparent similarity when comparing short segments of the profile with the entire sequence above a given threshold *D*_0_ (Fig 6b). Indeed, the transformation illustrated in Fig 6b resembles a renormalization-group transformation, analogous to those studied in the context of critical phenomena. In particular, the picture in the bottom panel of (Fig 6b) can be seen as a result of contracting the time axis by a factor *a* with respect to the middle panel, and successively decimating the number of events by the same factor. To demonstrate statistical self-similarity in the sequence of *θ*-bursts, we systematically analyze the functional form of the probability distributions *P*(Δ*t*; *D*_0_) for different thresholds *D*_0_ by rescaling each distribution on the average quiet time ${\langle \Delta t\rangle }_{D_{0}}$. Remarkably, we find that all distribution curves collapse onto a single functional form *G* (Fig 6c and 6d), defined by the following scaling relation

$$\begin{array}{c} P\left(\Delta t\right)={\left\langle \Delta t\right\rangle }^{-1}\cdot G\left(\Delta t/\left\langle \Delta t\right\rangle \right). \end{array}$$ (7)

The scaling relation in Eq 7 represents a quantitative, mathematical expression of the statistical self-similarity in the profile formed by the quiet times and *θ*-burst durations shown in Fig 6b. We find that the functional form *G* is well approximated by the generalized Gamma distribution $G\left(\Delta t/\langle \Delta t\rangle ;b,\nu ,p\right)=\left(p/b^{\nu }\right){\left(\Delta t/\langle \Delta t\rangle \right)}^{\nu -1}e^{{-(\Delta t/\langle \Delta t\rangle b)}^{p}}/\Gamma \left(\nu /p\right)$ [61], where in our analysis Δ*t*/〈Δ*t*〉 is a dimensionless quiet time. The Gamma functional form is homogeneous [62], i.e. rescaling the variable leaves the functional form unchanged. The existence of such scaling function indicates that the distribution of quiet times between consecutive *θ*-bursts is independent on the scale of observation *D*_0_. In the limit of *D*_0_ = 0, the quiet time distribution *P*(Δ*t*; *D*_0_) coincides with the distribution of *δ*-burst durations *P*_*δ*_ (Eq 2 and Figs 2b, 3b and 3d)—a Weibull functional form that belongs to the same class of homogeneous functions as the generalized Gamma.

Our data analysis shows that the scaling relation in Eq 7 and the associated Gamma functional form for the quiet times are robust: (i) we find it across the 24-hour sleep-wake cycle (Fig 6) as well as separately during light and dark periods (Fig F in S1 File), and (ii) it does not significantly change with lesion of the sleep-promoting neurons in the PZ brain area (compare Fig 6c and Fig 6d). Further, the presence of self-similar structure in quiet time indicates specific temporal order in the occurrence of *θ*-bursts. To explicitly verify this, we randomly reshuffle the data sequence of *θ*-burst durations, while preserving the *δ*-burst durations corresponding to quiet times at *D*_0_ = 0, and we perform the analysis on the reshuffled sequence to obtain quiet time distributions *P*_*rand*_(Δ*t*; *D*_0_) for different thresholds *D*_0_. After rescaling the distributions *P*_*rand*_(Δ*t*; *D*_0_) by the average quiet time ${\langle \Delta t\rangle }_{D_{0}}$, their curves collapse onto an exponential distribution (dashed lines in Fig 6c and 6d)—a hallmark of temporal independence between consecutive events [63]. This clearly demonstrates that temporal correlations are intimately related to the existence of universal non-exponential scaling functions (Eqs 2 and 7) [63, 64].

Notably, a similar temporal organization characterized by coexistence of power law and generalized Gamma distribution has been reported for active states and quiet times between them in a range of non-equilibrium systems self-tuning at criticality [15, 53, 55, 58]. Thus, our findings are a strong evidence in support of the hypothesis that bursting activity of fundamental brain rhythms and the associated sleep micro-architecture exhibit critical non-equilibrium behavior.

### Long-range scale-invariant correlations in *θ* and *δ* bursts

Physical systems at criticality exhibit high sensitivity to interactions among components [32, 65]. This leads to the emergence of collective cooperative behavior, where interactions span the entire system across space and time scales [32], leading to long-range correlations. Indeed, scaling features in such systems often arise in conjunction with long-range spatio-temporal correlations of power-law (scale-invariant) type, as observed at the critical point of continuous phase transitions [32]. Notably, physiological systems under neuroautonomic regulation also exhibit dynamics characterized by long-range power-law correlations—a scale-invariant structure that undergoes a phase transition with transitions from sleep to wake [66–69], with circadian rhythms [70–73] and under clinical conditions [74–76]. Further, the randomization procedure in the previous subsection (Fig 6) clearly demonstrates that a self-similar structure in quiet times, characterized by a Gamma scaling function (Eq 7), can arise only in the presence of a certain temporal order in *θ*-bursts. Thus, we next perform correlation analysis to quantify long-range features in the temporal organization of *δ*- and *θ*-burst durations.

**Fig 7:**
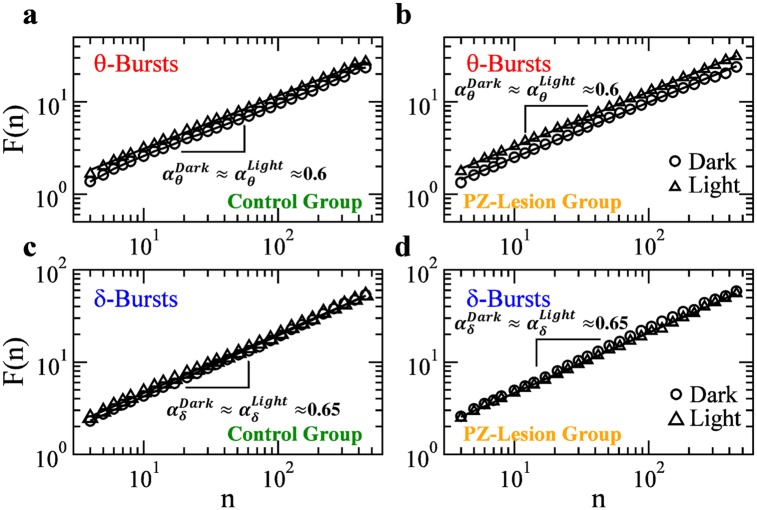
Long-range power-law correlations in sequences of consecutive *θ*- and *δ*-burst durations indicate a dynamical system at criticality. Detrended fluctuation analysis (Materials and methods: Data analysis) for sequences of *θ*- and *δ*-burst durations from control and PZ-lesioned rats. Burst durations are calculated for window size *w* = 5 s and threshold Th = 1 on the ratio *R*_*θδ*_ (Fig 1), and are analyzed separately for 12 h dark and light periods. The root mean square (r.m.s.) fluctuation function *F*(*n*) is obtained averaging over all rats in the control (a) and PZ-lesioned group (b), respectively. Log-log plots of *F*(*n*) vs the time scale of analysis *n*, where *n* is the number of consecutive burst durations, show power-law correlations over a broad range of scales *n*. The scaling exponents are significantly larger than 0.5, both in light and dark periods, indicating presence of positive (persistent) long-range correlations in *θ*-bursts for both control and PZ-lesioned rats. Similar results are found in sequences of *δ*-bursts for (c) control and (d) PZ-lesioned rats. The observed difference in the correlation exponents between *θ*- and *δ*-bursts is significant for both control and PZ rats during light and dark periods (*t*-test, *p* < 0.05).

To this end, we utilize the detrended fluctuation analysis (DFA)—a random walk based method, specially tailored to quantify long-range power-law correlations embedded in non-stationary signals with various polynomial trends and bursting dynamics [77, 78]. The DFA method is based on evaluation of the root mean square (r.m.s.) fluctuation function *F*(*n*) (Materials and methods, Data analysis), where *n* is the scale of analysis expressed in number of consecutive bursts (Fig 7). A scaling relationship of the form $F\left(n\right)\propto n^{\alpha_{d}}$ indicates presence of long-range power-law correlations in the time series of burst durations. Correlation exponent *α*_*d*_ ∈ [0, 0.5) indicates anti-correlations (where short burst durations tend to be followed by longer burst durations), while *α*_*d*_ ∈ (0.5, 1] indicates positive persistent correlations (long bursts tend to be followed by longer bursts)—a scale-invariant behavior that is consistent over several decades of time scale *n*; *α*_*d*_ = 0.5 corresponds to a random walk and absence of correlations.

We performed DFA on sequences of *θ*- and *δ*-burst durations separately, distinguishing between dark and light periods as well as between control and PZ-lesioned rats (Fig 7). We find that both *θ*- and *δ*-bursts exhibit long-range power-law correlations, with an exponent *α*_*d*_ = 0.60 ± 0.02 and *α*_*d*_ = 0.65 ± 0.02, respectively. These exponents are robust and do not change during dark or light periods, in line with our observations for the duration distributions (Fig 3). Moreover, our analyses indicate that PZ lesions do not affect the nature and strength of temporal correlations, and consistently shows that *θ*- and *δ*-bursts are long-range correlated across the sleep-wake cycle (Fig 7).

### Anti-correlated coupling between *δ*- and *θ*- bursts

Majority of physical and biological systems at equilibrium (homoeostasis) are controlled by mechanisms that either lead to dynamics with specific space or time scales characterized by exponential behaviors or to scale-invariant dynamics without characteristic scales following power laws. Non-equilibrium systems self-organizing at criticality are unique in the sense that they combine two distinct processes—a scale-invariant process related to the dynamics of active states and an exponential process related to quiet states—both of which emerge out of a single regulatory mechanism [13]. In that context, our findings of (i) power-law distribution for theta-burst durations (scale-invariant dynamics of active states) in coexistence with Weibull distribution for *δ*-burst durations (characteristic time scale for the dynamics of the quiet states) shown in Figs 2 and 3, (ii) universal Gamma distribution characterizing the temporal organization of quiet times across a range of scales (Fig 6), and (iii) long-range power-law correlations in the time sequence of *θ*- and *δ*-burst durations (Fig 7)—all these characteristics are typical for systems at criticality—indicate a common sleep regulatory mechanism for the bursting activity of both *θ* and *δ* rhythms and associated sleep micro-architecture. Presence of such common mechanism would imply coupling between *θ*- and *δ*-bursts. Indeed balanced excitation and inhibition in neuronal networks is essential to maintain critical-state dynamics [79–81]. Further, the concurrent change we find in both power-law and Weibull distribution parameters with transition from dark to light periods (Fig 3) is an additional indication of possible coupling between *θ*- and *δ*-bursts. Thus, we ask whether there is a cross-correlation between the durations of consecutive *δ*- and *θ*-bursts.

To further understand the temporal organization of bursting dynamics in relation to neuronal integrity in the PZ, we next investigate the coupling between consecutive *δ*- and *θ*-bursts, and the role of such coupling in the emergent scaling behavior of duration distributions in control and PZ-lesioned rats.

**Fig 8:**
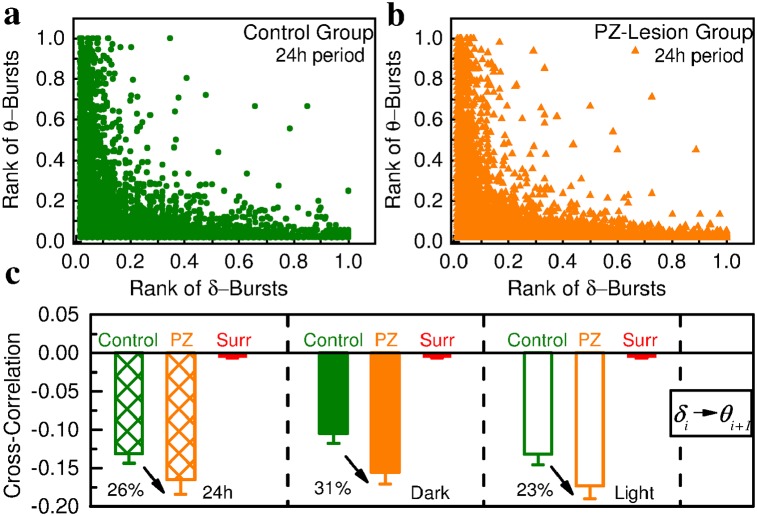
Coupling between *δ*- and *θ*-burst durations indicates a common mechanism regulating the activity of these rhythms in relation to sleep micro-architecture. Scatter plots and rank correlation analysis demonstrate coupling between consecutive *δ*- and *θ*-burst durations. (a) Scatter plot of *δ*-burst ranks vs following *θ*-burst ranks in the 24h period for control rats. Each dot represents a pair formed by a *δ*-burst and the following *θ*-burst, with burst durations separately ranked among the *δ*-bursts and the *θ*-bursts (longest duration corresponding to highest rank). (b) Scatter plot of *δ*-burst ranks vs following *θ*-burst ranks in the 24h period for PZ lesioned rats. For each rat group, ranks are calculated separately for each rat and then plotted together. (c) Average Spearman’s cross-correlation coefficient for control and PZ-lesioned rats in dark, light and 24h periods. Anti-correlations between consecutive *θ*- and *δ*-bursts are stronger during light than during dark periods in each of the two rat groups. Comparing dark vs light periods, the Student’s *t-test* gives *p* = 0.651 for control rats and *p* = 0.461 for PZ-lesioned rats. Importantly, PZ-lesioned rats generally exhibit stronger anti-correlations than the control group, in particular during dark periods, where the strength of anti-correlated coupling increases with ≈ 30% compared to control rats (control vs PZ *t-test*: 24h, *p* = 0.158; dark, *p* = 0.121; light, *p* = 0.064). All correlation coefficients calculated in both groups are significantly different from the corresponding values obtained in the surrogates (red bars) after randomly reshuffling the original order of *θ*- and *δ*-bursts (*t-test*: *p* < 0.001). All durations are calculated using a window *w* = 5 s and threshold *Th* = 1 on the ratio *R*_*θδ*_ (as in Fig 1). This finding of anti-correlated coupling between *θ*- and *δ*-bursts durations is further supported by an independent analysis based on conditional probability (Fig G in S1 File).

We first focus on the relationship between ranks of consecutive *δ*- and *θ*-burst durations, *d*_*δ*_ and *d*_*θ*_. We rank burst durations in ascending order, with the shortest duration corresponding to the smallest rank, and examine the scatter plots between the ranks of consecutive *d*_*δ*_ and *d*_*θ*_ (Fig 8a and 8b). We find that *δ*-bursts of high ranks (i.e. long durations) tend to be followed by *θ*-bursts of low ranks (i.e. short durations). This anti-correlated coupling is consistently present in both control (Fig 8a) and PZ-lesioned rats (Fig 8b), and appears to be a basic characteristic of dynamics as it is observed throughout the entire sleep-wake cycle in both dark and light periods.

To quantify the coupling between consecutive *δ*- and *θ*-burst durations we utilize Spearman’s correlation coefficient, which assesses monotonic relationships between two variables (Materials and methods, Data analysis). The Spearman’s cross-correlation is positive when observations of two variables have similar ranks, and negative if observations of two variables have opposite ranks. Our analyses show that the cross-correlation coefficient calculated for consecutive *δ*- and *θ*-burst durations is always (24h, dark, light period) significantly negative (Fig 8c)—a clear sign of anti-correlated coupling. This is verified by a surrogate test where the sequence of consecutive *δ*- and *θ*-burst durations is randomized (Fig 8c; Materials and methods, Data analysis). We find that the *δ*-*θ* anti-correlated coupling is more pronounced for PZ-lesioned rats, and this is consistently observed in both light and dark periods. Comparing light vs dark period, our results show an increase in the anti-correlated coupling during the light period within each group (Fig 8c).

The presence of anti-correlated coupling between consecutive *δ*- and *θ*-bursts is further supported by conditional probability analysis (Fig G in S1 File). Specifically, we ask how the conditional probability distribution $P(d_{\delta_{i}}|d_{\theta_{i-1}}>d^{*})$ of *δ*-burst durations $d_{\delta_{i}}$ depends on the length of preceding *θ*-burst duration $d_{\theta_{i-1}}$ above a given threshold d*—i.e., we consider the probability distribution of the subset of *δ*-bursts which follow *θ*-burst durations longer than d*. Note that when no condition is imposed on $d_{\theta_{i-1}}$, i.e. d* = 0, the conditional probability is equivalent to the Weibull distribution of *δ*-burst durations, namely $P\left(d_{\delta_{i}}|d_{\theta_{i-1}}>0\right)=P\left(d_{\delta_{i}}\right)$ (Figs 2 and 3). On the other hand, for threshold d* > 0, $P\left(d_{\delta_{i}}|d_{\theta_{i-1}}>d^{*}\right)\neq P\left(d_{\delta_{i}}\right)$ implies that $d_{\delta_{i}}$ and $d_{\theta_{i-1}}$ are not independent of each other. As discussed in further details in the Supplementary Information, we find that increasing the conditional threshold for $d_{\theta_{i-1}}$ the probability for longer $d_{\delta_{i}}$ significantly drops, while the probability for shorter $d_{\delta_{i}}$ significantly increases (Fig G insets in S1 File). This dependence is observed during light and dark periods for both control and PZ-lesioned groups, and clearly confirms the anti-correlated coupling between the durations of consecutive *δ*- and *θ*-bursts.

### Phenomenological model of coupling and criticality in *θ*- and *δ*-bursts dynamics

**Fig 9:**
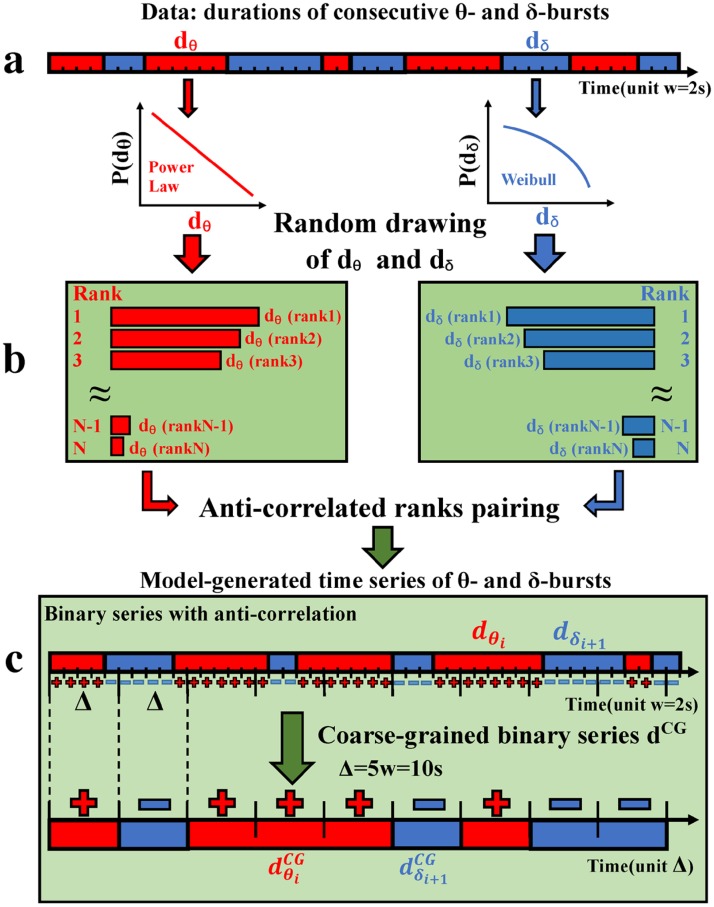
Schematic diagram of a phenomenological model to generate sequences of *θ*- and *δ*-burst durations with varied degree of anti-correlated coupling. (a) First, burst durations *d*_*θ*_ and *d*_*δ*_ are randomly drawn from the empirically obtained distributions (power law and Weibull, Fig 2) and separately ranked. Durations *d* = *n* * *w* are a multiple of the scale of analysis (window size *w* = 2 s). (b) Ranks of *θ*- and *δ*-burst durations are then paired to form an anti-correlated sequence: if the rank(*d*_*θ*_) of a *θ*-burst is large, than the rank(*d*_*δ*_) of the following *d*_*δ*_-burst is selected to be smaller, and vice versa. Repeating this process leads to a sequence of generated *d*_*θ*_ and *d*_*δ*_ durations with a certain degree of anti-correlation. (c) This newly generated anti-correlated time series is binarized, i.e. ‘+’/’-’ is assigned to each window *w* that belongs either to a *d*_*θ*_ (red, ‘+’) or *d*_*δ*_ (blue, ‘-’) duration, respectively. The binary time series is then coarse grained according to a majority rule applied over a window Δ = 5*w*. From the resulting coarse-grained (CG) binary series, consecutive *θ* durations, $d_{\theta }^{CG}$, and *δ* durations, $d_{\delta }^{CG}$, are extracted. Details of the model are given in Materials and methods: Model of anti-correlated burst coupling.

We next test whether the established anti-correlated coupling between consecutive *δ*- and *θ*-burst durations is essential for the emergent duality of power-law and Weibull distribution as a basic characteristic of systems at criticality. We develop a phenomenological model (Fig 9) based on anti-correlated pairing of *θ* and *δ* durations randomly drawn from the empirical distributions of the *δ*- and *θ*-burst durations established in our study (Fig 2). The model allows to control the degree of anti-correlations between consecutive *θ*- and *δ*-burst durations, and thus to examine if and how anti-correlations affect the emerging power-law and Weibull distributions of burst durations, and the related scale-invariant temporal structure of *θ*- and *δ*-bursts.

The basic steps to generate sequences of alternating *θ*- and *δ*-burst durations with the desired degree of correlations are schematically outlined in Fig 9 (Materials and methods, model of anti-correlated burst coupling.). Specifically: (1) we randomly draw durations *d*_*θ*_ and *d*_*δ*_ from their respective power-law and Weibull distributions obtained from our empirical data analyses (Fig 9a); (2) *d*_*θ*_ and *d*_*δ*_ are next separately sorted in ascending order, from shortest to longest, and are assigned a unique rank (Fig 9b); (3) the durations *d*_*θ*_ and *d*_*δ*_ are then paired based on their ranks with a certain degree of anti-correlation (defined by and monotonically depending on a single parameter) to generate an artificial time series of alternating *θ*- and *δ*-burst durations (Fig 9b); (4) the obtained artificial time series is then binarized in windows *w* corresponding to the window size used in our EEG spectral power analysis of the original data (Fig 1a), where ‘+’ is assigned for *θ*-bursts and ‘−’ for *δ*-bursts. Finally, the binary series is coarse-grained at larger time scale Δ (Fig 9c).

**Fig 10:**
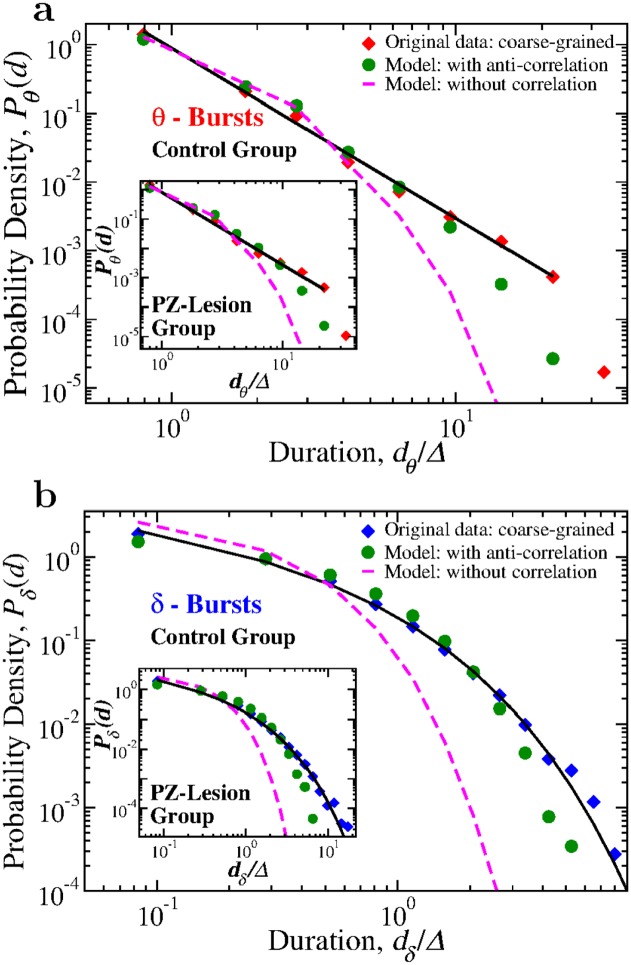
Anti-correlations between consecutive *θ*- and *δ*-bursts durations are essential for emerging critical behavior with duality of power-law and Weibull dynamics. Probability distributions of *θ*- and *δ*-burst durations from 24 h control and PZ-lesioned rat data coarse-grained (CG) over a window Δ = 10 s, are compared with the distributions obtained from the model-generated coarse-grained binary time series of *θ*- and *δ*-bursts durations with anti-correlations and without correlations (random pairing of *θ*- and *δ*-bursts, Fig 9). (a) Distributions *P*_*θ*_(*d*) of *θ*-burst durations for: (i) 24 h control rats data (red diamonds), (ii) model-generated time series of *θ*- and *δ*-bursts durations with anti-correlations (green circles), and (iii) model-generated time series with random pairing of *θ*- and *δ*-bursts durations (magenta dashed line). Inset shows results from same analysis on *P*_*θ*_(*d*) for the group of PZ-lesioned rats. (b) Distribution *P*_*δ*_(*d*) of *δ*-burst durations for: (i) 24h control rats data (blue diamonds), (ii) model-generated time series with anti-correlations (green circles), and (iii) model-generated time series with random pairing of *θ*- and *δ*-bursts durations (magenta dashed line). Inset shows results from same analysis on *P*_*δ*_(*d*) for the group of PZ-lesioned rats. In both (a) and (b), durations are in units of Δ, which is the window size used to coarse grain the sequences of *θ*- and *δ*-bursts durations. The distributions obtained from the model using anti-correlated *d*_*θ*_ and *d*_*δ*_ pairing (green circles) closely match the duration distributions for the original data (diamonds)—power law for *P*_*θ*_(*d*) and Weibull for *P*_*δ*_(*d*)—for both control and PZ-lesioned rats. In contrast, a random pairing of *d*_*θ*_ and *d*_*δ*_ produces duration distributions following the Poisson functional form (magenta dashed lines) that significantly deviates from the original data.

We utilize this model to test our hypothesis that coupling between *δ*- and *θ*-bursts is essential for the emergent duality of power-law and Weibull behavior across time scales as a hallmark of system at criticality. We test to what extent *δ*-*θ* coupling strength plays role in the emergent scale-invariant organization of sleep micro-architecture. Our simulations show that the generated distributions of *δ*- and *θ*-burst durations depend on the degree of anti-correlation introduced in the model. When the Spearman’s cross-correlation coefficient of burst durations generated by the model corresponds to the empirical values found in real data, the distributions obtained from the model approximate the empirical distributions (Fig 10), and scale-invariant temporal organization in burst durations emerges over a range of coarse-graining scales Δ. In contrast, absence of anti-correlated coupling in our model (i.e. random pairing of *δ* and *θ* durations in the generated time series) leads to exponential distribution for both *δ*- and *θ*-bursts, significantly different from real data (Fig 10).

## Discussion

We show that transient bursts in both the *θ* and *δ* cortical rhythms continuously occur during the sleep-wake cycle and exhibit a complex temporal organization that is invariant across a range of time scales, from seconds to minutes, and underlies sleep micro-architecture. We discover a remarkable duality of scale-invariant power-law distribution for *θ*-burst durations (active states) and a Weibull distribution with a exponential characteristic time scale for the *δ*-burst durations (quiet states) (Fig 2)—a behavior which is typically observed in non-equilibrium systems self-organizing at criticality, where a quiescent phase with exponential dynamics coexists with active events following power-law distributed sizes and durations [15, 34, 35, 57]. Further, we identify presence of coupling between *θ*- and *δ*-bursts dynamics which is characterized by significant anti-correlation in consecutive *θ*- and *δ*-burst durations (Fig 8), and we demonstrate through both empirical and modeling approaches that this anti-correlated coupling is essential part of the mechanism responsible for the emergent duality of power-law and Weibull behavior across time scales (Figs 9 and 10). Importantly, we find that sequences of consecutive *θ*- or *δ*-burst durations are long-range power-law correlated, indicating a scale-invariant organization in the temporal order of burst durations and a unique underlying process with persistent ‘memory’ spanning over a wide range of scales that statistically couples the duration of a given burst with the durations of hundreds of following bursts (Fig 7). Presence of complex temporal organization and coupling in cortical rhythms is also manifested through the self-similar structure we uncover in the quiet times separating consecutive *θ*-busts (active events) above a given duration (Fig 6)—described by a homogeneous generalized Gamma distribution [61]. This self-similar structure links, across time scales, the duration of a given *θ*-burst with the time of its occurrence.

Our empirical analyses show that the characteristics of *θ*- and *δ*-bursts dynamics do not depend on the scale of observation or on the threshold used to separate *θ*- from *δ*-bursts, and remain continuously present during dark and light periods (Figs 4, 5 and Figs B, C, D, E in S1 File), under different dominant physiologic states, and both in control rats and rats where all PZ neurons are lesioned. Thus, our findings indicate that the discovered scale-invariant organization in the bursting activity of *θ* and *δ* cortical rhythms is independent of behavioral factors across the sleep-wake cycle, and is not affected by the loss of neuronal inputs from the PZ, a major component of the sleep-promoting circuitry. Further, the presence of multiple scale-invariant characteristics related to distributions, correlations, coupling and timing of bursting events is a strong evidence of critical self-organization in *θ* and *δ* cortical rhythms—a signature of collective behavior over a range of time scales that emerges from neuronal interactions across brain areas which is essential to maintain system’s susceptibility and flexibility for abrupt sleep-stage and arousal transitions. The reported self-organization at the integrated cortical level, with dynamics spontaneously driven at criticality by active and quiet states, indicates that a non-equilibrium mechanism regulates sleep micro-architecture on time scales from seconds to minutes—a behavior which is in contrast to the homeostatic (equilibrium) regulation that controls consolidated sleep and wake, ultradian and circadian rhythms at large horizons of hours and days.

Our observations provide new insights about the role of PZ. Previous studies have demonstrated that PZ lesions cause a significant decrease of NREM sleep during the light period, without affecting the distribution of EEG spectral power among different brain rhythms [6, 51]. Our results suggest that such stability of the spectral power distribution results from the robust temporal organization and cross-talk of *δ* and *θ* rhythms, which seems not to be significantly altered by lesion of the PZ neurons. Furthermore, our findings show that the decrease in NREM sleep is not associated with significant changes in the dynamic characteristics and temporal organization of *δ* rhythms.

Sleep and wake are under control of complex regulatory circuitry involving multiple neuronal assemblies and specialized brain areas. GABAergic neurons from the PZ, together with other NREM sleep-promoting neurons, inhibit wake-promoting populations and arousal pathway which are crucial for cortical activation and wakefulness. During the past few years multiple NREM sleep promoting neuronal populations have been described [82–85]. Understanding the specific role of PZ sleep-promoting neurons and how they interact with other sleep- and wake-promoting brain areas to influence cortical activity is crucial to develop an integrated picture of sleep-wake control. For example, it is likely that other neuronal assemblies compensate for the loss of PZ neurons, e.g. the sleep-promoting neurons located in the ventrolateral preoptic area (VLPO) [86]. Our findings of scale-invariant features of *θ*- and *δ*-bursts dynamics which remain present after lesioning the PZ neurons, while at the same time total NREM sleep declines [6, 51], are a strong indication that the function of PZ neurons relates to sleep initiation, and may not be actively involved in regulating the dynamics once a sleep episode is initiated—i.e., lesioning the PZ reduces sleep initiation, and thus leading to decline in total NREM sleep; however, once sleep is initiated the dynamics and micro-architecture of *θ*- and *δ*-bursts are not significantly altered, raising the hypothesis that another sleep-promoting center (possibly the VLPO) may be involved in the dynamic aspects of sleep regulation. Moreover, our empirical findings of increased anti-correlation between durations of consecutive *θ*- and *δ*-bursts under PZ lesion (Fig 8) and model simulations demonstrating that such *θ*-*δ* coupling is essential for the emerging scale-invariant temporal organization in these cortical rhythms (Figs 9 and 10), indicate that PZ neurons may have dual role for both sleep and arousal/brief wake activation.

Importantly, the uncovered complex dynamics in *θ* and *δ* cortical rhythms do not depend on the time scales of observation, and share striking similarities with the dynamical characteristics of natural phenomena exhibiting non-equilibrium behavior of active and quiet states, and self-organization at criticality. Our analyses and findings are based on the interpretation of *θ*-bursts as active states and *δ*-bursts as quiet states, and on the hypothesis that the observed scale-invariant temporal organization of *θ* and *δ* rhythms emerges out of a common sleep regulatory mechanism, in analogy to the cooperative mechanisms underlying the dynamics of non-equilibrium systems at criticality. This approach is consistent with the basic neurophysiological understanding of *δ* rhythm as the quiet state cortical default mode—indeed, lesion and transection experiments have reported that interruption of sensory inputs to the cortex results in a cortical EEG similar to that in NREM sleep [87–89]. In contrast, oscillations in the *θ* band are associated with REM, arousals and wakefulness [10, 11]. Due to the respective amount of wakefulness and REM sleep in our data [50], most of the analyzed *θ*-bursts are likely associated with arousals and wake. Further, during wakefulness cortical *θ* activations are desynchronized with a wide range of burst durations, and correspondingly our analyses show that collective cortical neuronal activity follows robust universal scaling laws: power-law distribution for burst durations, long-range power-law correlations in the sequence of burst durations, and a self-similar structure in quiet times between bursts, all reminiscent of the dynamic characteristics and temporal organization of active states found in avalanche and earthquake dynamics [59, 60].

The presented findings provide a general picture unifying previous empirical observations of criticality in spontaneous brain dynamics at different levels—from networks of dissociated cortical neurons [90] and local field potentials (LFP) in cortex slice cultures [45], human EEG and fMRI resting state dynamics [48, 49, 91, 92], awake monkeys [93] and resting magnetoencephalography of the human brain [46], to the dynamics of sleep-stage and arousal transitions across species [13, 26, 27, 31, 94]—where either distributions or temporal correlations of active events have been studied and discussed in the context of self-organization at criticality. Several models based on statistical physics have also shown that the functional properties of the healthy brain resemble those of systems at criticality, and that altered physiologic states may correspond to super-critical or sub-critical states [95, 96]. In particular, models including disorder and frustration [47, 97]—essential features of spin glasses as well as of multi-layer cortical networks where neurons may be frustrated by receiving both excitatory and inhibitory inputs—provide critical exponents in good agreement with those observed experimentally depending on the balance between excitatory and inhibitory neurons and their distributions among cortical layers [81]. Crucially, here we show that important physiologic functions may benefit from underlying critical dynamics, and demonstrate presence of the full spectrum of scaling characteristics typical for non-equilibrium systems self-organizing at criticality. Furthermore, we link our observations to the collective behavior of a key sleep-promoting neuronal population leading to emerging cortical rhythms in relation to physiological alternation of sleep and wake. We find that the power-law scaling exponent of the distribution for *θ*-burst durations is *α* ≃ 2.35, a robust temporal organization in bursting activity across the sleep-wake cycle in both control and PZ lesioned rats. Notably, this scaling exponent is close to the power-law exponent *α* ≃ 2 for the distribution of neuronal avalanches in cortex slice cultures [45] and dissociated cortical neurons [90], to *α* ≃ 2.2 reported for arousal/wake episodes in coarse-grained sleep-stage recordings in humans [13, 31] and other species [26, 27, 30, 94].

In summary, our findings of scaling features for a full spectrum of dynamic characteristics in the bursting activity of cortical *θ* and *δ* rhythms strongly support the hypothesis of an underlying critical dynamics for sleep regulation [26, 65]. In systems far from equilibrium, emerging bursting activity described by power laws and exhibiting long-range spatio-temporal correlations has been proposed as an indication of self-organized criticality (SOC) [16, 17, 45]. In this context, bursts do not have a characteristic duration, and short as well as long bursts are expression of the same underlying dynamics [17]. Consecutive bursts are separated by quiescence periods whose distribution depends on the details of the system and generally exhibit an exponential tail [55, 58, 98, 99], and is an exponential for the paradigmatic sandpile model of SOC [17, 35]. Thus, in systems exhibiting self-organized criticality power-law and exponential dynamics for active and quiet states coexists, and emerge out of the same regulatory mechanism. The robust duality of power-law (scale invariant) and Weibull (exponential tail) distribution for the bursting dynamics of *θ* and *δ* rhythms is closely reminiscent of this scenario, where scale-free *θ*-bursts in cortical activity can be seen as avalanches or earthquakes [17, 55], while *δ*-bursts can be interpreted as the quiet periods between active states. Notably, the Weibull functional form for the distribution of quiet periods represents an extreme (minimal) event statistics. In the proposed here criticality framework of cortical rhythm dynamics and sleep micro-architecture, one can interpret the Weibull distribution as realization of shortest quiet periods between spontaneous initiation of active states (*θ*-bursts). Since such activations are spontaneously initiated at multiple brain locations, what one measures at the EEG probe is the closest (fastest arriving) activation. Following the analogy with SOC systems, we further demonstrated that the organization (occurrence in time) of *θ*-bursts is coupled with their durations, forming a scale-invariant structure for the quiet times between consecutive *θ*-burst above a given duration described by a universal Gamma distribution (also observed in earthquake dynamics [55]).

Overall, the combined empirical observations and modeling simulations reported here lay the foundation for a new paradigm for the investigation of sleep dynamics and sleep-stage transitions mechanisms, considering sleep micro-architecture as result of a non-equilibrium process and self-organization among neuronal assemblies to maintain a critical state—a behavior which is in stark contrast to the traditional homeostasis paradigm of sleep regulation at large time scales. Within this criticality paradigm arousals/brief wake and sleep-stage dynamics emerge out of a common regulatory mechanism, where arousals play an essential part in maintaining a non-equilibrium behavior at criticality, as evidenced by our observations of coupling between consecutive *θ*- and *δ*-bursts durations. Systems at criticality exhibit high susceptibility and sensitivity to interactions, leading to cooperative behaviors over a range of space/time scales, and thus, maintaining a critical state through self-organization is important for system’s flexibility and for generating spontaneous transitions [100, 101]. Such transitions can not occur intrinsically in a homeostatic (equilibrium) systems. In the context of sleep micro-architecture, the proposed criticality-based paradigm may provide new insights on the origin and mechanisms underlying the dynamics of sleep-stage and arousal transitions, and offers a unifying picture of sleep and wake.

## Materials and methods

### Experimental setup

#### Animals

Pathogen-free adult male Sprague Dawley rats (Harlan; 275-300 g; n = 20) were used in this study. Care of these animals in the experiments met National Institutes of Health standards, as set forth in the Guide for the Care and Use of Laboratory Animals, and all protocols were approved by the Beth Israel Deaconess Medical Center and Harvard Medical School Institutional Animal Care and Use Committees.

#### Surgery

For details on brain injection and implantation for polysomnographic recording in rats, see Anaclet et al., 2012 [50]. To perform cell-specific lesions, 10 rats received brain microinjections of 0.1% anti-orexin-B IgG saporin (OX-SAP; 130-330 nl; Advanced Targeting Systems) within the PZ [AP, -10.3 mm; L, ±2.1 mm; DV, -6.6 mm, as per the rat atlas of Paxinos and Watson (2005)]. The rat control group included five rats that received saline brain injections and five rats without brain injection. After the brain injections, the rats were implanted with polysomnographic electrodes. Four electroencephalogram (EEG) screw electrodes (Plastics One, stock # E363/20/4.8/SP) were implanted into the skull, in the frontal (2) and parietal bones (2) of each side. Two flexible electromyogram (EMG) wire electrodes (Plastics One, stock # E363/76) were placed in the neck muscles. After EEG/EMG implantation, rats were single housed until the end of the experiments.

#### Sleep-wake recording

Ten days after surgery, the rats were moved in an insulated soundproofed recording chamber maintained at an ambient temperature of 22 ± 1 C and on a 12-h light/dark cycle (lights-on at 7 a.m.) with food and water available ad libitum. They were connected via flexible recording cables and a commutator (Plastics One, stock # 363-363) to an analog amplifier (A-M Systems, Model 3500) and computer, with an analog-to-digital converter card and running Vital Recorder (KISSEI COMTEC CO., LTD., Japan). After 3-5 day habituation period, EEG/EMG were recorded for 48 h, beginning at 7:00 P.M. Cortical EEG and EMG signals were amplified and digitalized with a resolution of 256 Hz. Recordings used in this study have already been subjected to sleep-wake analysis and published [50].

#### Histology

At the end of the experiments, rats were perfused under deep anesthesia (200 mg per kg of chloral hydrate) with 50-ml saline, followed by 200-ml of neutral phosphate-buffered formalin (4%, vol/vol, Fischer Scientific). After perfusion, the brains were removed, postfixed in neutral phosphate-buffered formalin for 2 hr, equilibrated in PBS containing sodium azide (0.02%) (PBS-azide) and sucrose (20%) for at least 1 day, and then sectioned at 40 *μ*m on a freezing microtome into four series. For verification of OX-SAP-induced brain lesions, one series of tissue was processed for Nissl staining as done previously (Lu et al., 2000). PZ lesion for each rat has previously been published (see Fig. 2 in [50]).

### Data recording and analysis

Ten control rats and ten rats with PZ lesion were used for this study. Cortical EEG signals were recorded continuously for 48 h from the left and right hemisphere with a resolution of 256 Hz. Two electrodes, one frontal and one parietal, were placed on each hemisphere. Cortical EEG is the differential potential between a frontal electrode (for *δ* frequencies) and a parietal electrode (for *θ* frequencies). The parietal electrode picks up *θ* rhythm from the hippocampus during REM sleep; both the frontal and parietal electrodes pick up *θ* rhythm during wakefulness. Hippocampal *θ* rhythm can be also present during wakefulness, mainly during cognitive wakefulness. The signal analyzed in this study is the difference between frontal and parietal EEG electrode potentials (*frontal* − *parietal* EEG) from one hemisphere (ipsilateral).

### Data pre-processing

EEG recordings were first normalized to zero mean, *μ* = 0, and unit standard deviation, *σ* = 1. For each rat, EEG signals were visually inspected and noisy segments were discarded. Only clean segments were finally included in the analysis.

#### Data filtering

Data were bandpass filtered in the range 0.5 − 25 Hz using a FIR (Finite Impulse Response) filter designed in Matlab.

### Data analysis

#### Spectral analysis

The clean EEG signal is divided in *N* non-overlapping windows of size *w* and the spectral power in the *δ* band (0.5 − 4 Hz), *S*_*δ*_, and in the *θ* band (4 − 8 Hz), *S*_*θ*_, is estimated in each window using Welch’s method [102]. The analysis is performed for several values of the window size *w*, from 2 s to 10 s. Results are generally independent of *w*, as shown in Fig 5, as well as Fig D and Fig E in S1 File, and extensively discussed in the main text.

#### *θ*- and *δ*-burst detection and definition

The ratio *R*_*θδ*_ = *S*_*θ*_/*S*_*δ*_ between *θ* and *δ* power is calculated in each window *k*, with *k* = 1, 2, …, *N*, and a time series *R*_*θδ*_(*k*) is obtained. Given a threshold *Th* ≥ 1, a *θ*-burst is defined as a sequence of *n* consecutive windows where *R*_*θδ*_ > *Th*, while a *δ*-burst consists in a sequence of *n* consecutive windows where *R*_*θδ*_ < 1/*Th* (Fig 1). The duration of a burst is given by *d* = *n* ⋅ *w*. Durations of *θ* (*δ*) bursts are denoted by *d*_*θ*_ (*d*_*δ*_). The threshold *Th* is set equal to 1 throughout the analysis. Results are independent of *Th*, as shown in Fig 4, as well as Fig B and Fig C in S1 File, and extensively discussed in the main text.

#### Surrogate test for *θ*- and *δ*-burst duration distributions (Fig A in S1 File)

For each rat, the time series *R*_*θδ*_(*k*) is randomly reshuffled to obtain a surrogate $R_{\theta \delta }^{*}\left(k^{\prime }\right)$. Surrogate *θ*- and *δ*-bursts durations are then calculated from $R_{\theta \delta }^{*}\left(k^{\prime }\right)$ following the procedure illustrated in the previous paragraph. The corresponding *θ*- and *δ*-burst duration distributions are shown in the Fig A in S1 File, together with distributions from original data.

#### Definition of quiet time Δ*t*

A quiet time Δ*t* is defined as the time interval between the ending time of a burst $t_{j}^{e}$ and the starting time $t_{j+1}^{s}$ of the following one, namely $\Delta t_{j}=t_{j+1}^{s}-t_{j}^{e}$.

#### Data binning

Probability distributions of *θ*-burst durations are calculated using logarithmic binning, i.e. linear binning in logarithmic scale. Denoting a set of bin boundaries as *B* = (*b*_1_, *b*_2_, …, *b*_*k*_) and fixing *b*_1_ = 0.5*w*, the logarithmic bins fulfill the relation *b*_*i*+1_ = *b*_*i*_ ⋅ 10^*c*^, which implies that the bin size is constant in logarithmic scale, i.e. *logb*_*i*+1_ − *logb*_*i*_ = *c*. The following bin size *c* have been used in this study: Figs 2, 3, 5, Fig D and Fig E in S1 File, *c* = 0.2; Fig 4, 10, Fig B and Fig C in S1 File, *c* = 0.18.

Probability distributions of *δ*-burst durations are calculated using the following binning procedure. Given a window size *w*, the bin boundaries *e*_1_, *e*_2_, …, *e*_*n*_, …, *e*_*k*_ are obtained using the recursive relation $e_{n}=e_{1}+w\sum_{i=2}^{n}b^{i-1}$, with *e*_1_ = 0.5*w* and *n* ≥ 2. The following values of the parameter *b* have been used in this study: Fig 6, Fig G in S1 File, *b* = 1.6; All other figures, *b* = 1.2.

#### Fit of burst duration distributions

Power-law exponents are estimated by fitting the *θ*-burst duration distributions with the functional form *log*(*P*(*d*)) = −*αlog*(*d*)+ *C*, using the least square method. The Weibull parameters are estimated by fitting the observed duration distribution to *P*(*d*) = (*β*/λ)(*d*/λ)^*β*−1^*exp* − (*d*/λ)^*β*^, utilizing the Levenberg-Marquardt non-linear least squares algorithm [103], where the error variance at *P*(*d*) is estimated as *MSE*(1/*W*), where *W* = 1/*P*_*δ*_(*d*)^2^ is the weight.”

#### Error bars

Error bars *δP* on each value of the distribution presented in Figs 2 and 3 are given by

$$\begin{array}{c} \delta P=\frac{1}{dD}\sqrt{\frac{p(1-p)}{N}}, \end{array}$$ (8)

where *p* = *P*(*D*)*dD* is the probability to observe a duration *D* in the range [*D*, *D* + *dD*] and *N* is the total number of bursts and *dD* is the corresponding bin size.

#### Spearman’s correlation

Given to variables *X* and *Y*, the Spearman’s correlation coefficient is defined as

$$\begin{array}{c} \rho_{s}=\frac{cov(rg_{X},rg_{Y})}{\sigma_{rg_{X}}\sigma_{rg_{Y}}}, \end{array}$$ (9)

where *rg*_*X*_ and *rg*_*Y*_ are the tied rankings of *X* and *Y* [104], respectively, $\sigma_{rg_{X}}$ and $\sigma_{rg_{Y}}$ their standard deviations, and *cov*(*rg*_*X*_, *rg*_*Y*_) indicates the covariance between *rg*_*X*_ and *rg*_*Y*_.

#### Surrogate test for correlations between consecutive *θ*- and *δ*-burst duration

To test significance of correlations between consecutive *θ*- and *δ*-burst durations, a surrogate sequence of burst durations is generated for each rat by randomly reshuffling the original order of *θ*- and *δ*-bursts. The Spearman’s correlation coefficient *ρ*_*s*_ between consecutive *θ*- and *δ*-bursts is calculated for each surrogate. The average Spearman’s correlation coefficient obtained from all surrogates is then compared with the average correlation coefficient calculated from the original sequences of burst durations via *t* − *test* (Section Results, Fig 8). Correlation coefficients for surrogate data for both control and PZ-lesioned rats during dark, light and 24h are all with value |*ρ*_*s*_| < 10^−3^.

#### Detrented Fluctuations Analysis (DFA)

The DFA is a method based on random walk [105]. It improves the classical fluctuation analysis (FA) for non-stationary signals where embedded polynomial trends mask the intrinsic correlation properties in the fluctuations [105]. The performance of DFA for signals with different types of non-stationarities and artifacts has been extensively studied and compared to other methods of correlation analysis [78, 106–108]. The DFA method is briefly described by the following steps [105]:

1. A given signal *u*_*i*_ (*i* = 1, …, *N*, where *N* is the length of the signal) is integrated to obtain $y\left(k\right)\equiv \sum_{i=1}^{k}[u\left(i\right)-\langle u\rangle ]$, where 〈*u*〉 is the mean of *u*_*i*_.
2. The integrated signal *y*(*k*) is divided into boxes of equal length *n*.
3. In each box of length *n* we fit *y*(*k*) using a first order polynomial function which represents the trend in that box. The *y* coordinate of the fit curve in each box is denoted by *y*_*n*_(*k*).
4. The integrated profile *y*(*k*) is detrended by subtracting the local trend *y*_*n*_(*k*) in each box of length *n*
  $$\begin{array}{c} Y\left(k\right)\equiv y\left(k\right)-y_{n}\left(k\right) \end{array}$$ (10)
5. For a given box length *n*, calculate the root-mean-square (r.m.s.) fluctuation function for this integrated and detrended signal
  $$\begin{array}{c} F\left(n\right)\equiv \sqrt{\frac{1}{N}\sum_{k=1}^{N}{\left[Y\left(k\right)\right]}^{2}} \end{array}$$ (11)
6. Repeat the above computation over a broad range of box lengths *n*, where *n* represents a specific space or time scale, to obtain a functional relationship between *F*(*n*) and *n*.

For a power-law correlated time series, the average r.m.s. fluctuation function *F*(*n*) and the box size *n* are connected by a power-law relation, that is $F\left(n\right)∼n^{\alpha_{d}}$. The exponent *α*_*d*_ is a parameter which quantifies the long-range power-law correlation properties of the signal. Values of *α*_*d*_ < 0.5 indicate the presence of anti-correlations in the time series, *α*_*d*_ = 0.5 absence of correlations (white noise), and *α*_*d*_ > 0.5 indicates the presence of positive correlations in the time series.

### Conditional probability distributions

The conditional probability of an event *H* for a given event *X* is defined as

$$\begin{array}{c} P\left(H|X\right)=\frac{P(H\cap X)}{P(X)}, \end{array}$$ (12)

where *P*(*H* ∩ *X*) is the probability that *H* and *X* jointly occur, and *P*(*X*) > 0 is the probability of the event *X*. The condition *X* reduces the statistics and increases the fluctuations of the distribution *P*(*H*|*X*) as compared to *P*(*H*). For this reason one associates the following error to each bin of the densities [56]:

$$\begin{array}{c} \epsilon_{H}=\frac{1}{dH}\sqrt{\frac{p(1-p)}{N}}, \end{array}$$ (13)

where *p* = *P*(*H*)*dH* is the probability to observe a *H* in the range [*H*, *H* + *dH*] and *N* is the total number of events.

It is a basic result of probability theory that *P*(*H*|*X*) = *P*(*X*) if and only if *H* does not depend on *X*. On the contrary, *P*(*H*|*X*) ≠ *P*(*X*) implies that *H* and *X* are not independent of each other, and their relation can be quantified by a suitable correlation measure. In the analysis of burst coupling, *H* and *X* are considered significantly correlated if *P*(*H*|*X*) − *P*(*X*) > *ϵ*_*H*_.

To obtain a detailed picture of temporal correlations and coupling between consecutive bursts, we investigated how the individual *δ*-burst durations are influenced by the preceding *θ*-bursts. To this end, we evaluated the conditional probability density $P(d_{\delta }^{i}|X\left(i-1\right))$ for different conditions *X*(*i* − 1) on preceding burst durations $d_{\theta }^{i-1}$ (Fig G in S1 File). If $d_{\delta }^{i}$ is independent of preceding bursts, then $P\left(d_{\delta }^{i}|X\right)=P\left(d_{\delta }\right)$. On the other hand, $P\left(d_{\delta }^{i}|X\left(i-1\right)\right)\neq P\left(d_{\delta }^{i}\right)$ implies that the duration $d_{\delta }^{i}$ depends on the property of the preceding bursts specified by the condition *X*(*i* − 1). We focused on the conditions $d_{\theta }^{i-1}>d^{*}$, and evaluated the conditional probability $P(d_{\delta }^{i}|d_{\theta }^{i-1}>d^{*})$ for different d*. The analysis of the conditional probability distribution $P(d_{\delta }^{i}|d_{\theta }^{i-1}>d^{*})$ for different d* values clearly confirms the existence of *θ*/*δ* anti-correlated coupling across the entire sleep/wake cycle (Fig G in S1 File). Selecting only longer preceding *θ*-bursts, the probabilities for long lasting *δ*-bursts systematically decrease, while very short *δ*-bursts become more and more likely (Fig G in S1 File). Importantly, the analysis of conditional probabilities shows that increasing the threshold d*, i.e. progressively retaining only longer and longer preceding *θ*-bursts, produces a selective increase only in the probabilities of very short *δ*-bursts, a feature that could not be captured by the Spearman’s correlation coefficient.

### Model of anti-correlated burst coupling

The model consists of the following steps.

#### Random drawing and ranking

*N* durations *d*_*θ*_ and *d*_*δ*_ are randomly drawn from the empirical distributions previously obtained using a specific window size *w*. *d*_*θ*_ and *d*_*δ*_ are separately sorted in ascending order, i.e. from shortest to longest, and get a distinct ordinal numbers from *k* = 1, 2, …, *N*, which corresponds to their rank. This procedure ensures that each duration has a unique rank. The ranked *d*_*θ*_ and *d*_*δ*_ are then paired with a tunable degree of anti-correlation and a new time series of alternating *θ*- and *δ*-burst durations is thus generated. The coarse-grained properties of the resulting time series depends on the degree of anti-correlations used in the pairing.

#### Correlated pairing

Once *d*_*θ*_ and *d*_*δ*_ are ranked and a distinct, unique ordinal number is associated to them, one randomly choose a *d*_*θ*_ with rank *k*_1_ between 1 and *N*. To choose the following *d*_*δ*_, one draws a random number *k*_2_ from a Gaussian distribution with mean *μ* = 1 + *N* − *k*_1_ and standard deviation *σ*, and takes *d*_*δ*_ as the duration corresponding to rank *k*_2_. This procedure is iterated *N* times, and at each iteration *i* the mean of the Gaussian from which one draws the next random rank, *k*_*i*_, depends on *k*_*i*−1_, i.e. *μ* = 1 + *N* − *k*_*i*−1_. At each iteration, *k*_*i*_ will correspond to a duration *d*_*δ*_ from the sorted *δ*-burst durations if the preceding burst was a *θ*-burst with duration *d*_*θ*_, whereas *k*_*i*_ will select a duration *d*_*θ*_ from the sorted *θ*-burst durations if the preceding burst was a *δ*-burst with duration *d*_*δ*_. As a result one obtains a sequence of *d*_*θ*_ and *d*_*δ*_ whose degree of anti-correlations is controlled by a single parameter, *σ*. The smaller *σ*, the stronger anti-correlations are.

#### Binary series and coarse-graining

To characterize the coarse-grained properties, the time series is first converted in a binary sequence, namely a sequence of ‘+’ and ‘-’. Since each duration is by definition a multiple *n* of the unit window *w*, namely *d* = *nw*, the *n* windows belonging to a *d*_*θ*_ are populated with ‘+’, while the *n* windows belonging to a *d*_*δ*_ with ‘-’ (Fig 9c). As a results one has a sequence of windows populated with ‘+’ and ‘-’. This binary sequence is then coarse-grained grouping a given number Δ of consecutive windows, with Δ odd number, and assigning ‘+’ or ‘-’ to the new windows of size Δ according to a majority rule, i.e. one assign ‘+’ (’-’) if the number of ‘+’ is larger (smaller) than the number of ‘-’ (Fig 9c). A Coarse-Grained Binary Sequence (CGBS) is thus obtained, and *d*^*CG*^ coarse-grained durations are calculated as shown in Fig 10.

## Supporting information

S1 File Supporting information presenting tests to validate reported empirical results for recordings during 12-hour light and 12-hour dark periods and for different parameter values: (i) surrogate test to confirm physiological origin of the power-law distribution for *θ*-burst and Weibull distribution for *δ*-burst durations (Fig. A); (ii) range of threshold values Th for the ratio *R*_*θδ*_(*t*) between *δ* and *θ* spectral power (Fig. B and Fig. C); (iii) different window sizes *w* for the EEG spectral power analysis (Fig. D and Fig. E); (iv) different threshold values *D*_0_ defining quite times between consecutive *θ*-bursts to test for scale-invariant organization in quiet times (Fig. F); and (v) conditional probabilities *P*(*d*_*δi*_ | *d*_*θi*−1_) of *δ*-burst durations *d*_*δi*_ for different values of the preceding *θ*-burst duration *d*_*θi*−1_ to confirm robustness of anti-correlated coupling between consecutive *δ*- and *θ*-bursts (Fig. G).
(PDF)
