# Supplementary material for: Non-equilibrium critical dynamics of bursts in θ and δ rhythms as fundamental characteristic of sleep and wake micro-architecture
Source: PLoS Comput Biol. 2019 Nov 14;15(11):e1007268. doi: 10.1371/journal.pcbi.1007268 (PMC6855414; doi:10.1371/journal.pcbi.1007268)
Supplement: S1 File — Supporting information presenting tests to validate reported empirical results for recordings during 12-hour light and 12-hour dark periods and for different parameter values: (i) surrogate test to confirm physiological origin of the power-law distribution for θ-burst and Weibull distribution for δ-burst durations (Fig. A); (ii) range of threshold values Th for the ratio Rθδ(t) between δ and θ spectral power (Fig. B and Fig. C); (iii) different window sizes w for the EEG spectral power analysis (Fig. D and Fig. E); (iv) different threshold values D0 defining quite times between consecutive θ-bursts to test for scale-invariant organization in quiet times (Fig. F); and (v) conditional probabilities P(dδi | dθi−1) of δ-burst durations dδi for different values of the preceding θ-burst duration dθi−1 to confirm robustness of anti-correlated coupling between consecutive δ- and θ-bursts (Fig. G). (PDF) [file pcbi.1007268.s001.pdf]

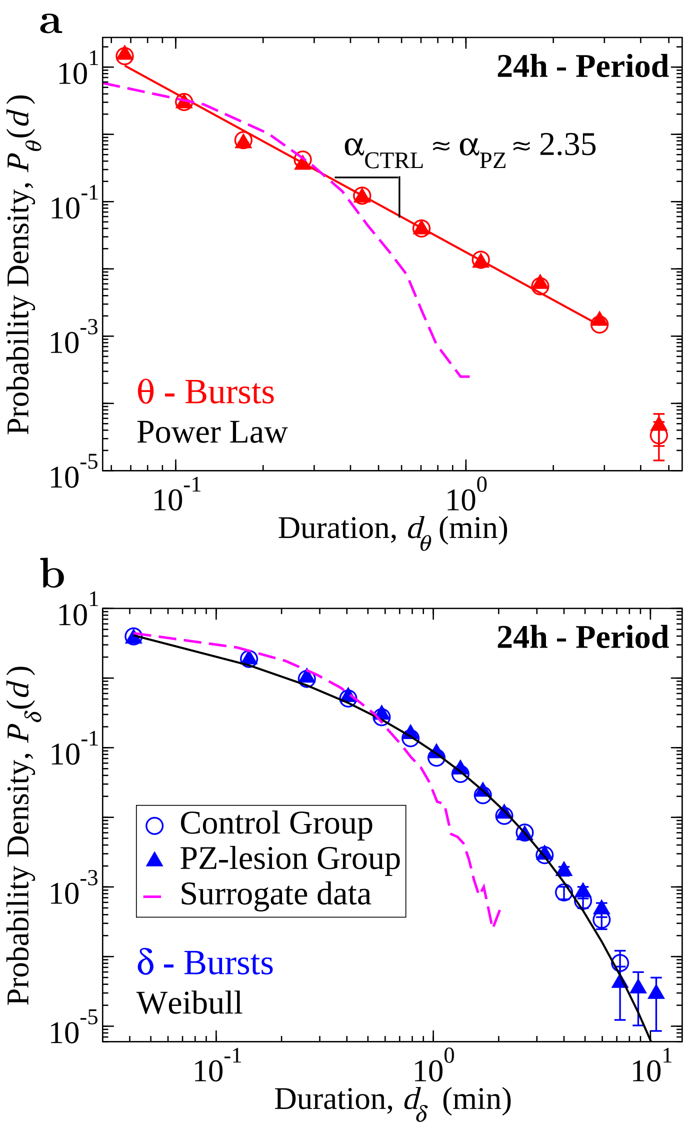

Fig. A **Distributions of surrogate θ- and δ-burst durations markedly deviates from the original distributions discussed in the main text (Fig. 2).** Procedure for generating surrogate θ- and δ-burst durations is explained in the Materials and methods, Data analysis. (a) Probability density of original θ-burst durations for control (open circles) and PZ-lesioned rats (full triangles) over the 24h period (pooled data) are compared with the probability density of surrogate θ-burst durations (magenta dashed line). In contrast to the original θ-burst durations, the probability density of surrogates does not follow a power-law behavior, and is close to an exponential distribution. (b) Probability density of original δ-burst durations for control (open circles) and PZ-lesioned rats (full triangles) over 24 h period (pooled data) are compared with the probability density of surrogate δ-burst durations (magenta dashed line). The probability density of surrogate δ-burst durations departs from the original Weibull and follows an exponential behavior.

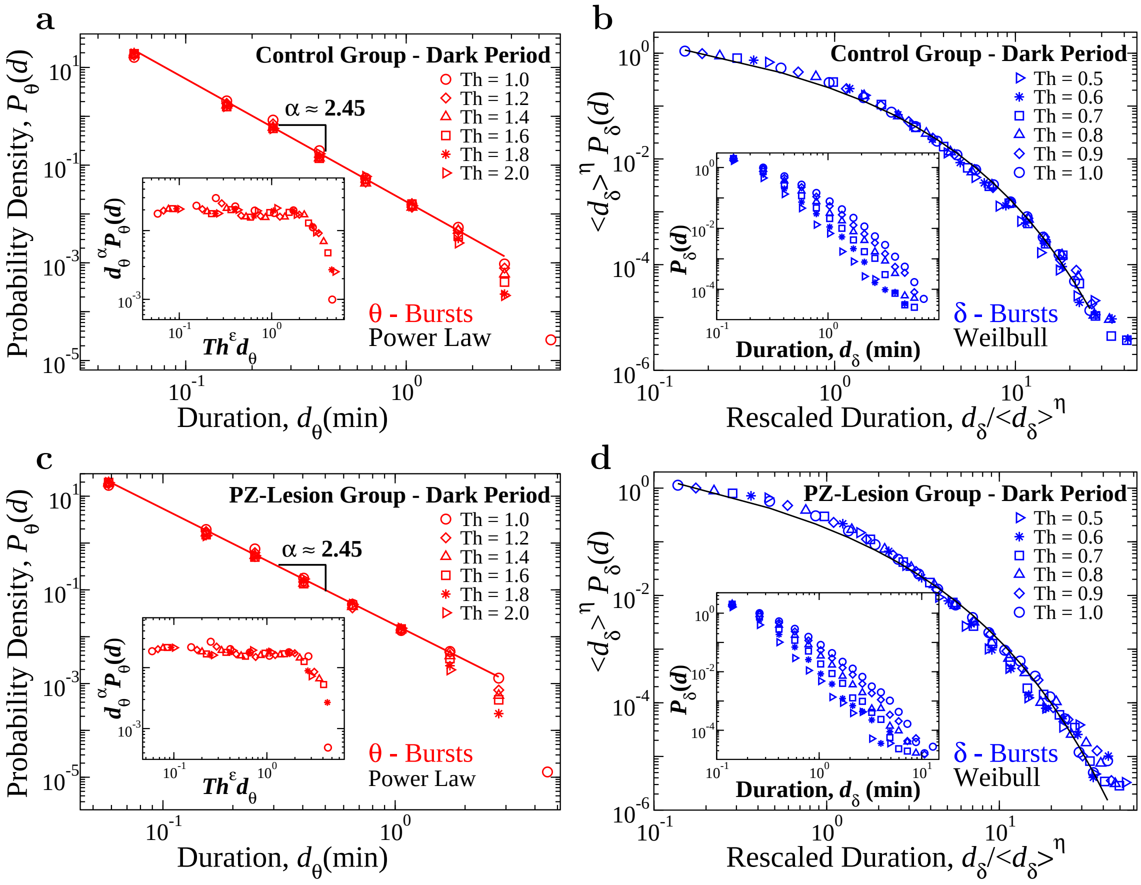

Fig. B **Distribution of θ- and δ-burst durations in the dark period are independent of the specific threshold Th used to identify bursts and can be described by unique scaling functions.** Distribution of θ- and δ-burst durations in the dark period for different threshold values Th on the ratio $R_{\theta\delta}$ and window size $w = 5$ s. (a) Distribution of θ-burst durations for control rats over a 12 h dark period (pooled data). Distributions evaluated using different Th values consistently follow the same power-law behaviour (red tick line), with an exponential cutoff that is controlled by Th. Inset: the data collapse onto a universal function f_θ when we plot $P(d)d^\alpha$ versus $Th^\epsilon d$, with $\alpha = 2.45$ and $\epsilon = 0.8$. (b) Rescaled distribution of δ-burst durations for control rats over a 12 h dark period (pooled data). Distributions are rescaled by $\langle d_\delta \rangle^\eta$, with $\langle d_\delta \rangle$ mean δ-burst duration and $\eta = 1.2$. After rescaling, distributions collapse onto a single function that is well fitted by a Weibull distribution $f(d; \lambda, \beta)$ (black thick line), with $\lambda = 0.60$ and $\beta = 0.54$. Inset: distributions P_δ for different thresholds Th (not rescaled). (c) Distribution of θ-burst durations for PZ-lesioned rats over a 12h period (pooled data). Distributions evaluated using different Th values consistently follow the same power-law behavior (red tick line), with a cutoff that is controlled by Th. Inset: Data collapse onto a single function by plotting $P(d)d^\alpha$ versus $Th^\epsilon d$ with $\alpha = 2.45$ and $\epsilon = 0.8$. (d) Rescaled distribution of δ-burst durations for PZ-lesioned rats over a 12h dark period (pooled data). Distributions are rescaled by $\langle d_\delta \rangle^\eta$, with $\langle d_\delta \rangle$ mean δ-burst

duration and $\eta = 1.2$. After rescaling, the distributions collapse onto a single function that is well fitted by a Weibull distribution $f(d; \lambda, \beta)$ (black thick line), with $\beta = 0.50$ and $\lambda = 0.37$. Inset: Distributions P_δ for different thresholds (not rescaled).

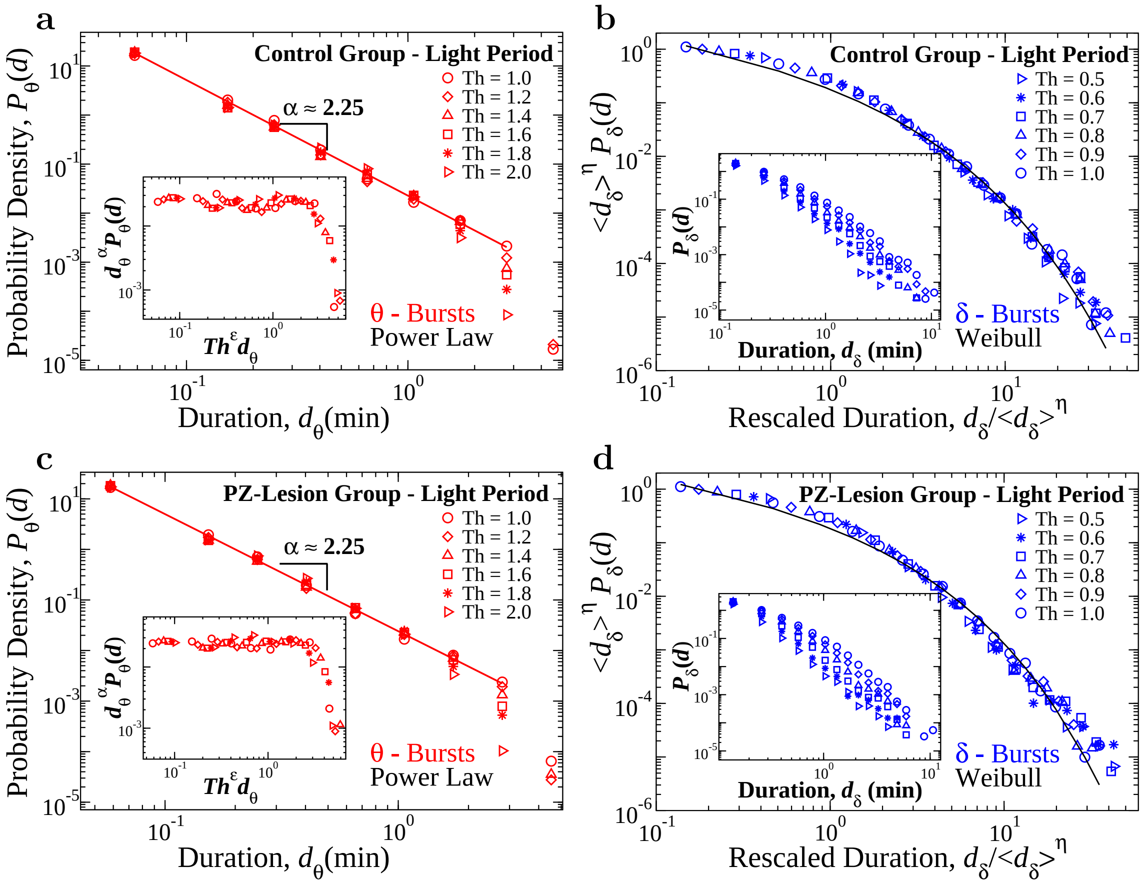

Fig. C **Distribution of θ- and δ-burst durations in the light period are independent of the specific threshold Th used to identify bursts and can be described by scaling functions.** Distribution of θ- and δ-burst durations in the light period for different threshold values Th on the ratio $R_{\theta\delta}$ and window size $w = 5$ s. (a) Distribution of θ-burst durations for control rats over a 12 h light period (pooled data). Distributions evaluated using different Th values consistently follow the same power-law behaviour (red tick line), with a cutoff that is controlled by Th. Inset: the data collapse onto a single function when we plot $P(d)d^{\alpha}$ versus $Th^{\epsilon}d$, with $\alpha = 2.25$ and $\epsilon = 0.8$. (b) Rescaled distribution of δ-burst durations for control rats over a 12 h light period (pooled data). Distributions are rescaled by $\langle d_\delta \rangle^{\eta}$, with $\langle d_\delta \rangle$ mean δ-burst duration and $\eta = 1.2$. After rescaling, distributions collapse onto a single function, a Weibull distribution $f(d;\lambda,\beta)$ (black thick line), with $\beta = 0.55$ and $\lambda = 0.46$. Inset: Distributions P_δ for different thresholds (not rescaled). (c) Distribution of θ-burst durations for PZ-lesioned rats over a 12 h period (pooled data). Distributions evaluated using different Th values consistently follow the same power-law behaviour (red tick line), with a cutoff that is controlled by Th. Inset: data collapse onto a universal function f_θ by plotting $P(d)d^{\alpha}$ versus $Th^{\epsilon}d$ with $\alpha = 2.25$ and $\epsilon = 0.8$. (d) Rescaled distribution of δ-burst durations for PZ-lesioned rats over a 12 h light period (pooled data). Distributions are rescaled by $\langle d_\delta \rangle^{\eta}$, with $\langle d_\delta \rangle$ mean δ-burst duration and $\eta = 1.2$. After rescaling, the distributions

collapse onto a single function, a Weibull distribution $f(d;\lambda,\beta)$ (black thick line), with $\beta = 0.56$ and $\lambda = 0.47$. Inset: Distributions P_δ for different thresholds (not rescaled).

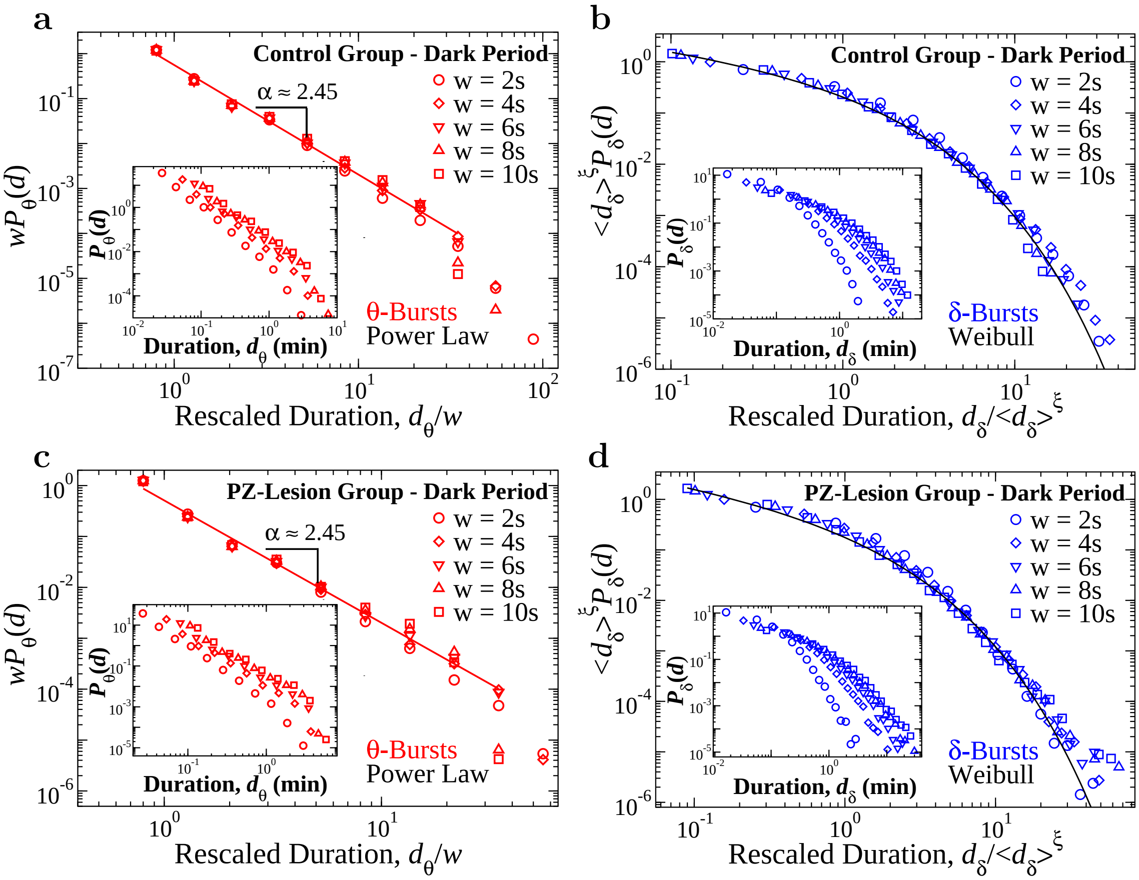

Fig. D **Distribution of θ- and δ-burst durations in the dark period are independent of the specific window size w used to identify bursts and can be described by unique scaling functions.** Distribution of θ- and δ-burst durations in dark period for different window sizes w and $Th = 1$ on the ratio $R_{\theta\delta}$. (a) Rescaled distribution of θ-burst durations for control rats over a 12h dark period (pooled data). Distributions are rescaled by the window size w and consistently show the same power-law behavior with $\alpha = 2.45$ (red tick line), as proven by the data collapse. Inset: Distributions P_θ for different window sizes (not rescaled). (b) Rescaled distribution of δ-burst durations for control rats over a 12 h dark period (pooled data). Distributions are rescaled by $\langle d_\delta \rangle^\xi$, with $\langle d_\delta \rangle$ mean δ-burst duration and $\xi = 1.2$, and collapse onto a single function that is well described by a Weibull distribution $f(d; \lambda, \beta)$ (black thick line), with $\beta = 0.59$ and $\lambda = 0.57$. Inset: Distributions P_δ for different window sizes (not rescaled). (c) Rescaled distribution of θ-burst durations for PZ-lesioned rats over a 12 h dark period (pooled data). Distributions are rescaled by the window size w and consistently show the same power-law behavior with $\alpha = 2.45$ (red tick line), as proven by the data collapse. Inset: Distributions P_θ for different window sizes (not rescaled). (d) Rescaled distribution of δ-burst durations for PZ-lesioned rats over a 12h dark period (pooled data). Distributions are rescaled by $\langle d_\delta \rangle^\xi$, with $\langle d_\delta \rangle$ mean δ-burst duration and $\xi = 1.2$, and collapse onto a single function that is well fitted by a Weibull distribution

$f(d; \lambda, \beta)$ (black thick line), with $\beta = 0.54$ and $\lambda = 0.45$. Inset: Distributions P_δ for different window sizes (not rescaled).

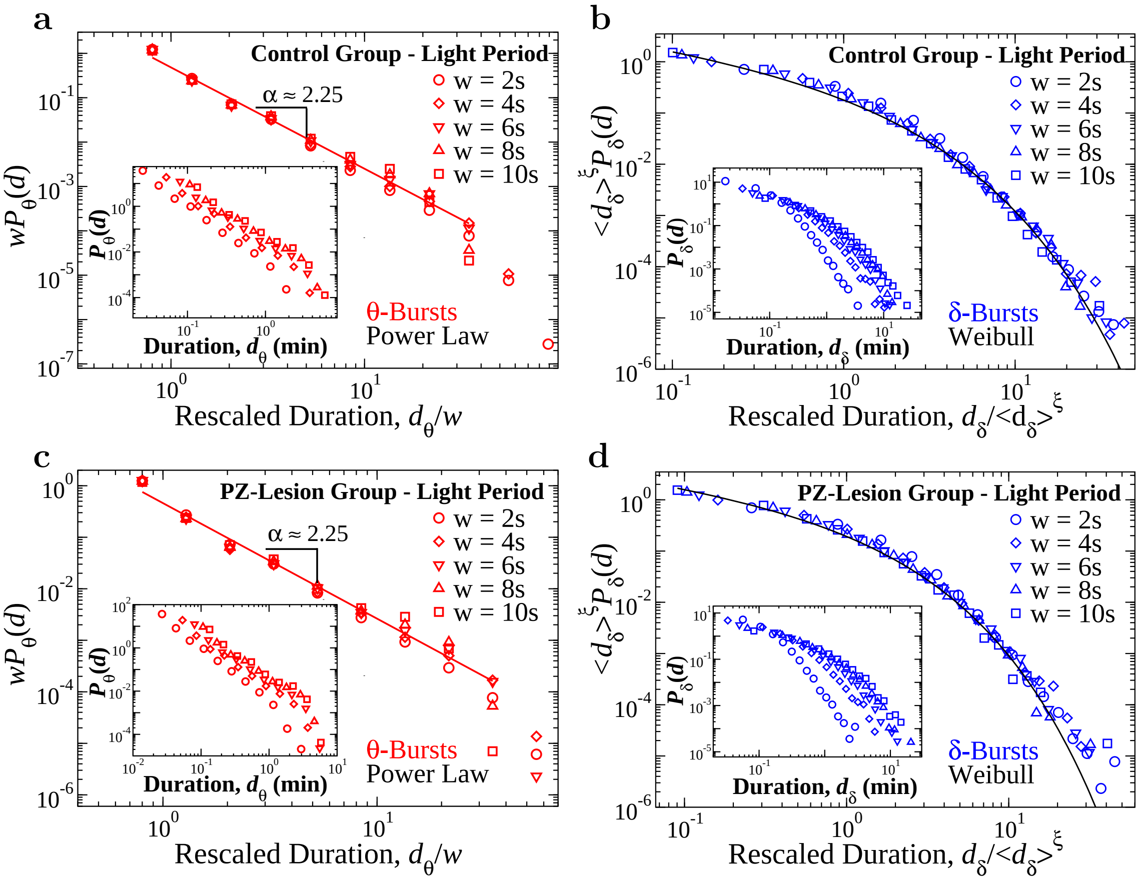

Fig. E **Distribution of θ- and δ-burst durations in the light period are independent of the specific window size w used to identify bursts and can be described by unique scaling functions.** Distribution of θ- and δ-burst durations in light period for different window sizes w and $Th = 1$ on the ratio $R_{\theta\delta}$. (a) Rescaled distribution of θ-burst durations for control rats over a 12h light period (pooled data). Distributions are rescaled by the window size w and consistently show the same power-law behavior with $\alpha = 2.25$ (red tick line), as proven by the data collapse. Inset: Distributions P_θ for different window sizes (not rescaled). (b) Rescaled distribution of δ-burst durations for control rats over a 12h light period (pooled data). Distributions are rescaled by $\langle d_\delta \rangle^\xi$, with $\langle d_\delta \rangle$ mean δ-burst duration and $\xi = 1.2$, and collapse onto a single function that is well described by a Weibull distribution $f(d; \lambda, \beta)$ (black thick line), with $\beta = 0.54$ and $\lambda = 0.44$. Inset: Distributions P_δ for different window sizes (not rescaled). (c) Rescaled distribution of θ-burst durations for PZ- lesioned rats over a 12h light period (pooled data). Distributions are rescaled by the window size w and consistently show the same power-law behavior with $\alpha = 2.25$ (red tick line), as proven by the data collapse. Inset: Distributions P_θ for different window sizes (not rescaled). (d) Rescaled distribution of δ-burst durations for PZ-lesioned rats over a 12 h light period (pooled data). Distributions are rescaled by $\langle d_\delta \rangle^\xi$, with $\langle d_\delta \rangle$ mean δ-burst duration and $\xi = 1.2$, and collapse onto a single function that is well described by a

Weibull distribution $f(d; \lambda, \beta)$ (black thick line), with $\beta = 0.56$ and $\lambda = 0.48$. Inset: Distributions P_δ for different window sizes (not rescaled).

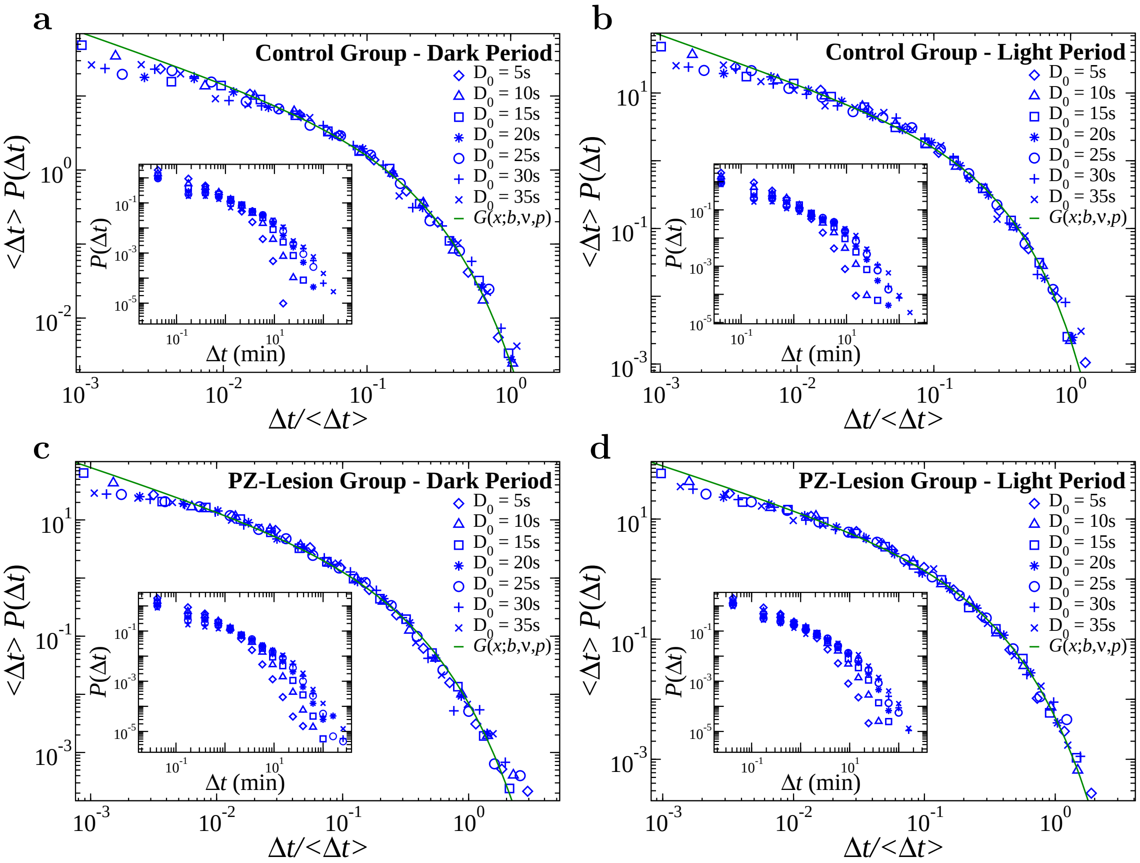

Fig. F **Distribution of quiet times between consecutive θ-bursts for different thresholds D_0 on θ-burst durations in dark and light periods. When rescaled by $\langle \Delta t \rangle$ (main panels), distributions collapse onto a unique function that is well fitted by a generalized Gamma function $G(x; b, \nu, p)$ (green tick lines).** (a) Distributions of quiet times for different thresholds D_0 on θ-burst durations in 12h dark period for control rats. Generalized gamma fit (green tick line): $b = 1$, $\nu = 0.58$, and $p = 0.8$. (b) Distributions of quiet times for different thresholds D_0 on θ-burst durations in 12 h light period for control rats. Generalized gamma fit (green tick line): $b = 1$, $\nu = 0.47$, and $p = 0.77$. (c) Distributions of quiet times for different thresholds D_0 on θ-burst durations in 12 h dark period for PZ-lesioned rats. Generalized gamma (green tick line): $b = 0.95$, $\nu = 0.47$, and $p = 0.72$. (d) Distributions of quiet times for different thresholds D_0 on θ-burst durations in 12 h light period for PZ-lesioned rats. Generalized gamma fit (green tick line): $b = 1.89$, $\nu = 0.35$, and $p = 0.93$.

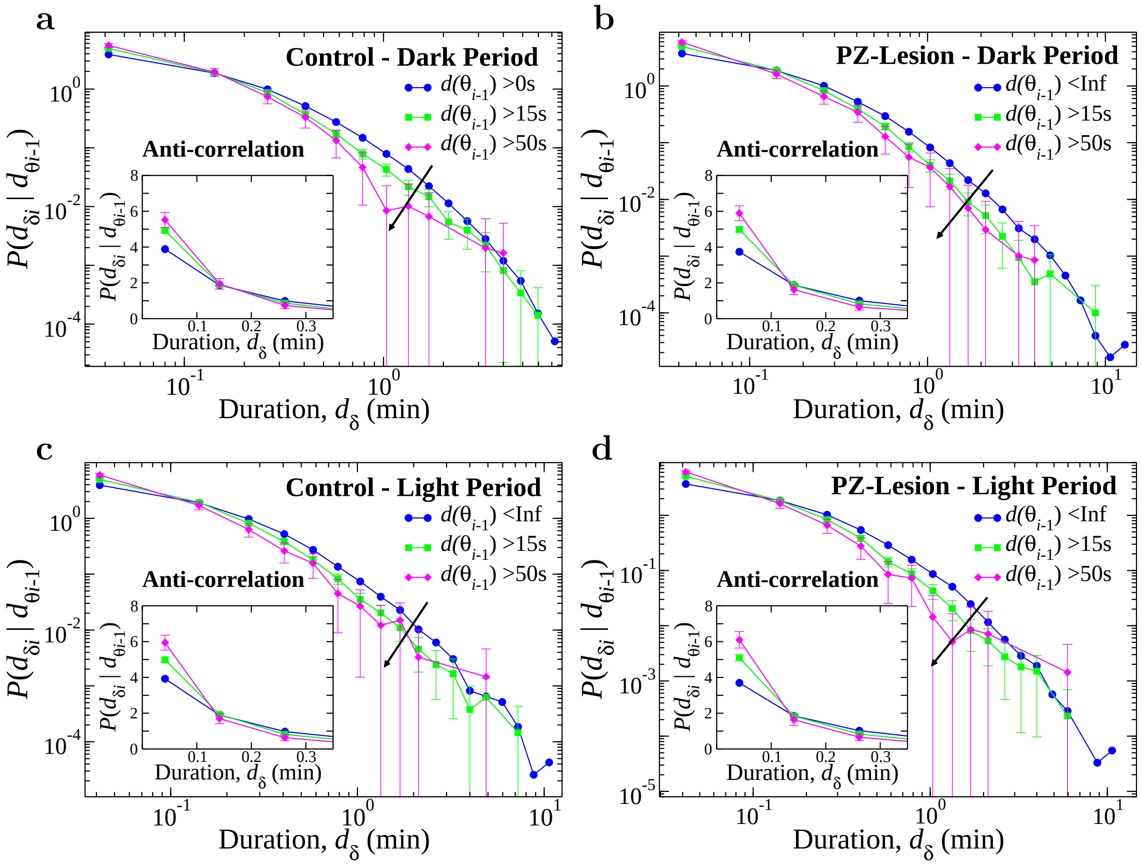

Fig. G **Distributions of conditional probabilities for δ-burst durations.** (a) Distribution of durations d_{δ_i} given that the duration of the preceding θ-burst is larger than a given threshold d^* ($d^* = 15$s and $d^* = 50$s) for control rats in dark periods. (b) Distribution of durations d_{δ_i} given that the duration of the preceding θ-burst is larger than a given threshold, $d^* = 15$s and $d^* = 50$s, for PZ-lesioned rats in dark periods. Durations are calculated using a window $w = 5$ s and a threshold $Th = 1$ on the ratio $R_{\theta/\delta}$. (c) Distribution of durations d_{δ_i} given that the duration of the preceding θ-burst is larger than a given threshold d^* ($d^* = 15$s and $d^* = 50$s) for control rats in light periods. (d) Distribution of durations d_{δ_i} given that the duration of the preceding θ-burst is larger than a given threshold, $d^* = 15$s and $d^* = 50$s, for PZ-lesioned rats in light periods. Durations are calculated using a window $w = 5$ s and a threshold $Th = 1$ on the ratio $R_{\theta\delta}$. Inset in each panel shows details of conditional probability distribution for short $d_{\delta_i} < 0.4$ min.
